# SMRT Sequencing of Paramecium Bursaria Chlorella Virus-1 Reveals Diverse Methylation Stability in Adenines Targeted by Restriction Modification Systems

**DOI:** 10.3389/fmicb.2020.00887

**Published:** 2020-05-19

**Authors:** Samantha R. Coy, Eric R. Gann, Spiridon E. Papoulis, Michael E. Holder, Nadim J. Ajami, Joseph F. Petrosino, Erik R. Zinser, James L. Van Etten, Steven W. Wilhelm

**Affiliations:** ^1^Department of Microbiology, The University of Tennessee, Knoxville, Knoxville, TN, United States; ^2^BioSciences at Rice, Rice University, Houston, TX, United States; ^3^Alkek Center for Metagenomics and Microbiome Research, Baylor College of Medicine, Houston, TX, United States; ^4^Department of Plant Pathology and the Nebraska Center for Virology, University of Nebraska, Lincoln, NE, United States

**Keywords:** NCLDV, chloroviruses, PBCV-1, algal-virus, DNA methylation, hemimethylation, restriction modification

## Abstract

Chloroviruses (family *Phycodnaviridae*) infect eukaryotic, freshwater, unicellular green algae. A unique feature of these viruses is an abundance of DNA methyltransferases, with isolates dedicating up to 4.5% of their protein coding potential to these genes. This diversity highlights just one of the long-standing values of the chlorovirus model system; where group-wide epigenomic characterization might begin to elucidate the function(s) of DNA methylation in large dsDNA viruses. We characterized DNA modifications in the prototype chlorovirus, PBCV-1, using single-molecule real time (SMRT) sequencing (*aka* PacBio). Results were compared to total available sites predicted *in silico* based on DNA sequence alone. SMRT-software detected N6-methyl-adenine (m6A) at GATC and CATG recognition sites, motifs previously shown to be targeted by PBCV-1 DNA methyltransferases M.CviAI and M. *Cvi*AII, respectively. At the same time, PacBio analyses indicated that 10.9% of the PBCV-1 genome had large interpulse duration ratio (ipdRatio) values, the primary metric for DNA modification identification. These events represent 20.6x more sites than can be accounted for by all available adenines in GATC and CATG motifs, suggesting base or backbone modifications other than methylation might be present. To define methylation stability, we cross-compared methylation status of each GATC and CATG sequence in three biological replicates and found ∼81% of sites were stably methylated, while ∼2% consistently lack methylation. The remaining 17% of sites were stochastically methylated. When methylation status was analyzed for both strands of each target, we show that palindromes existed in completely non-methylated states, fully-methylated states, or hemi-methylated states, though GATC sites more often lack methylation than CATG sequences. Given that both sequences are targeted by not just methyltransferases, but by restriction endonucleases that are together encoded by PBCV-1 as virus-originating restriction modification (RM) systems, there is strong selective pressure to modify all target sites. The finding that most instances of non-methylation are associated with hemi-methylation is congruent with observations that hemi-methylated palindromes are resistant to cleavage by restriction endonucleases. However, sites where hemi-methylation is conserved might represent a unique regulatory function for PBCV-1. This study serves as a baseline for future investigation into the epigenomics of chloroviruses and their giant virus relatives.

## Introduction

Viruses infecting eukaryotic algae play a critical role in aquatic ecosystems. Their lytic activity results in redistribution of organic matter from a particulate pool into a dissolved to particulate continuum that can be assimilated by the remaining microbial community, thus providing ecosystem stability and driving biodiversity (Thingstad and Lignell, 1997; Weinbauer and Rassoulzadegan, 2004; Anesio and Bellas, 2011; Martiny et al., 2011; Weitz et al., 2015). In the oceans, this activity is modeled to account for the daily, virus-driven cycling of up to a quarter of the total organic carbon in the surface oceans (Wilhelm and Suttle, 1999).

Although viruses are the most abundant entities in aquatic systems, only a small number of eukaryotic algal viruses have been isolated and are maintained in labs (Coy et al., 2018; Short et al., 2018). This group represents diverse nucleic acid types, architectures, and sizes, but the majority of isolates to date have large genomes made of double-stranded (ds) DNA. A phylogenetic comparison of these large viruses places them into the proposed monophyletic order, the *Megavirales* (Colson et al., 2013), also more commonly known as “giant” viruses or nuclear cytoplasmic large DNA viruses (NCLDVs). Giant viruses have long interested researchers for not only their size, often overlapping with that of cellular organisms (Wilhelm et al., 2017), but also for their unique genomic content. Indeed, these entities often encode central components of protein translation and DNA repair. Of interest to this study is an unusually high number of DNA methyltransferases. For instance, viruses that infect freshwater *Chlorella*-like green algae encode up to 18 distinct DNA methyltransferases, representing up to ∼4.5% of their total protein coding potential (Chan et al., 2006; Fitzgerald et al., 2007). Many of these enzymes share only distant homology to DNA methyltransferases encoded by cellular organisms, and thus may represent enzymes uniquely adapted for viral functions.

DNA methyltransferases catalyze the transfer of a reactive methyl group from the common cellular metabolite, S-adenosyl-methionine, to form either methylated cytosine (m4C, m5C) or methylated adenine (m6A). Most known enzymes have evolved to modify cytosines or adenines in specific nucleotide sequences that are recognized by the enzyme. These sites often range from two to six base-pairs, though there are examples of more complicated and promiscuous recognition sequences (Roberts et al., 2015). In any case, the direct consequence of DNA methylation is a change in the primary and secondary structure of the DNA (Nathan and Crothers, 2002), which can influence a variety of DNA recognition and protein binding interactions.

DNA methylation is known to regulate a diverse number of physiological processes. The most well studied examples include a silencing role in gene expression, and as a component of bacterial restriction modification systems. However, several novel functions that are directed by DNA methylation have been recently identified (Casadesus and Low, 2006; Wion and Casadesus, 2006; Murphy et al., 2013), suggesting this is a major regulatory molecule for a variety of DNA-protein activities. Given this modification is found in many algal-infecting giant viruses, we suspect that it is used to confer functions not previously known to promote virus fitness. Before this question can be investigated, however, it is necessary to quantify the distribution and stability of DNA methylation in some of the model virus systems.

The chloroviruses are the model system for studying giant, algal-infecting viruses. Isolated over 35 years ago, this system has expanded to include several hundred isolates acquired across the globe (Jeanniard et al., 2013). The number of DNA methyltransferases encoded by each chlorovirus aligns well with the amount of methylation that is measured in its genome, ranging from 0.12 to 47.5% and 0 to 37% of cytosines and adenines, respectively (Van Etten et al., 1985; Van Etten et al., 1991). Previous studies have determined that the prototype chlorovirus, PBCV-1, exhibits methylation in 1.86% of cytosines (m5C) and 1.45% of adenines (m6A) (Van Etten et al., 1985), and that these modifications arise from the activity of five putative DNA methyltransferases, although one of the m5C methyltransferases is a pseudogene (Zhang et al., 1992a; Table 1). Target sequences of the two m6A decorating methyltransferases, CviAI (Xia and Van Etten, 1986) and *Cvi*AII (Zhang et al., 1992b) have been previously identified; they create G^m6^ATC and C^m^ATG sequences, respectively. These two enzymes are predicted to methylate all adenines occurring in these sequences of PBCV-1, which amounts to about 1.6% of the total genomic adenines. That previous measurements were slightly less than this suggests that maybe not all GATC and CATG sites are methylated. This perceived incomplete methylation represents a potential regulatory function for the virus, and thus warrants a higher resolution analysis of DNA methylation in the PBCV-1 genome. Moreover, because PBCV-1 has been subjected to genomic, transcriptomic, and proteomic studies (Yanai-Balser et al., 2010; Dunigan et al., 2012; Blanc et al., 2014), there is a rich biological context from which to assess its “epigenomic” state.

**TABLE 1:** Characteristics of DNA methyltransferases encoded by PBCV-1.

Name	Gene	Mod	Motif	Txc^a^	Notes
M.CviAI	*A581R*	m6A	GATC	E	Part of viral RM system (with *A579L*) (Xia et al., 1986; Xia and Van Etten, 1986)
M. *Cvi*AII	*A251R*	m6A	CATG	E	Part of viral RM system (with *A252R*) (Zhang et al., 1992b)
M.CviAIV	*A530R*	m5C	RGCB	L	Pseudogene, non-functional (Zhang et al., 1992a)
M.CviAIII	*A517L*	m5C	–	E	Putative short G + C rich sequence (Zhang et al., 1992a)
M.CviAV	*A683L*	m5C	–	EL	Putative m5C gene, function unconfirmed

*^a^Transcriptional status reprinted from Supplementary Tables in Dunigan et al. (2012). E, Early; L, Late; EL, Early-Late.*

In this study, we characterized the PBCV-1 DNA m6A methylation pattern and stability by contrasting total available sites determined *in silico* with site specific measurements using single-molecule real time (SMRT) sequencing (*aka* PacBio). This technique allowed us to determine the methylation status of each target site, as well as its stability across space (i.e., separate virus populations) and time (i.e., multiple generations). During sequencing, we also identified signatures of DNA modifications other than methylation and predict that these putative modifications might bear important consequences for viral infectivity. This study establishes a baseline for future investigation into DNA modifications in chloroviruses and serves as a framework for initiating these studies in other algal-infecting giant viruses.

## Materials and Methods

### Distribution of Methyltransferases Encoded by Viruses

To identify DNA methyltransferases in publicly available viral genomes, we chose a strategy that uses both BLAST 2.7.1 + and HMMER 3.1b2 to generate alignments to a reference database derived from experimentally characterized “Gold Standard” DNA methyltransferases found in New England Biolabs’ REBASE (Roberts et al., 2015). To identify functional motifs of DNA methyltransferases, we used hmmscan with gathering cutoffs to collect Pfams (from release 31) (El-Gebali et al., 2019) represented in our Gold Standard database, including PF05869.11 (Dam), PF00145.17 (DNA_methylase), PF07669.11 (*Eco*57I), PF13651.6 (*Eco*RI_methylase), PF12161.8 (HsdM_N), PF02086.15 (MethyltransfD12), PF02384.16 (N6_Mtase), PF01555.18 (N6_N4_Mtase), and PF12564.8 (TypeIII_RM_meth). Gold Standard methyltransferases that did not contain one of the Pfams used in this study were queried in BLAST searches to identify putative DNA methyltransferases in viral proteomes and can be found in “BLASTexceptions.fasta.” A viral protein was considered to be a DNA methyltransferase if its protein profile aligned with hmmsearch exceeded gathering cutoffs or the viral protein aligned to a DNA methyltransferase sequence *via* BLAST with the query protein being at least 75% of the alignment length and the *e*-value was < 1E-5.

Viral assembly metadata from RefSeq, GenBank, and NCBI taxonomy were downloaded on June 26th, 2018 and stored in “Viral_assemblydat.tsv” and “nodes.dmp.” If a virus was included in both RefSeq and GenBank, the RefSeq assembly was preferentially used as a query. Because viral assemblies deposited in GenBank are annotated using non-standardized approaches, we chose to translate all frames in these viral genomes to avoid inconsistencies in annotation approaches. DNA methyltransferase pfams and characterized methyltransferase sequences lacking pfams (outlined above) were queried with HMMER and BLAST, respectively, to annotate putative DNA methyltransferases found in the translated frames of each viral genome. To map viruses to the hosts they infect, mappings were downloaded from the Virus-Host DB^1^. Jupyter notebooks and associated source code used for DNA methyltransferase annotation can be found at www.github.com/SEpapoulis/MTannotation.

### PBCV-1 *in silico* Analysis of DNA Modification Predictions

The frequency of GATC and CATG sequences in the PBCV-1 genome was determined using sequence independent and dependent approaches. For the former, “SeqIndyEn.py” was used to evaluate the enrichment of PBCV-1 DNA methyltransferase target motif sequences. Given PBCV-1 has a G + C content of 40%, this yields an expected frequency of 0.2 for G/C and 0.3 for A/T, making GATC and CATG sequences likely to occur once every 278 base-pairs (1/[0.2^∗^0.3^∗^0.3^∗^0.3]). We calculated an enrichment score for GATC and CATG frequency based on the number of observed motifs in a 278 base-pair window subtracted from an expected occurrence of each motif (n-2). Next, we used “SeqIndyDep.py” to locate genomic regions that are depleted in the motif sites independent of sequence context. This was done by counting the length of sequence between two neighboring motifs, dividing by 278, and multiplying the quotient by 2 to account for both GATC and CATG sequences. Combined with the results from SeqIndyEn.py, this approach allowed us to define fold enrichment > 1 and fold depletion < 1. All scripts described here are available at www.github.com/SEpapoulis/MTannotation.

While the sequence independent approach is commonly used to define motif frequency, it is difficult to delineate what is selecting for enrichment or depletion of a DNA motif. Since protein-coding requirements are amongst the strongest selective forces on DNA sequence, we decided to analyze GATC and CATG frequency based on codon flexibility using the open source, on-line software DistAMo [27]. This software scores motif frequency on the basis of codon redundancy. For example, if a GATC motif exists in a query, but it could be replaced with a different four base-pair sequence to yield the same codon, then that GATC sequence is scored as enriched. On the other hand, if a sequence could be substituted with GATC to yield the same codon, but that GATC sequence does not exist in the query, then that region is scored as depleted. This approach applies a z-score to genes and/or defined window sizes, with scores greater than the absolute value of 2 marked as significant.

### Preparation of Viral DNA for SMRT Sequencing

Three batch cultures of *Chlorella variabilis* (NC64A) were grown in Modified Bold’s Basal medium at 25°C under continuous light (30 μmol photons m^–2^ s ^–1^) and gentle shaking (∼150 rpm) (Dunigan and Agarkova, 2016a). Virus PBCV-1 was added to *C. variabilis* NC64A during mid-logarithmic growth (μ = 1.22 ± 0.06/day) at an M.O.I. of ∼5 (Supplementary Figure S1). Cultures were incubated at conditions described above until the host population had visibly completely lysed, which occurred after 3 days.

Cell lysates were extracted in triplicate 30 ml samples using a phenol-chloroform method that selectively extracts viral DNA (Dunigan and Agarkova, 2016b). Viral DNA quality and quantity were assessed spectrophotometrically with a NanoDrop 1000 (Thermo Fisher Scientific, Inc., DE, United States). DNA was visualized using agarose gel electrophoresis to confirm the presence of high molecular weight DNA (>40 kb), and subjected to a PCR screen for 16S rRNA gene targets to monitor cellular contamination. Extracted DNA was stored at −20°C prior to sequencing.

### SMRT Sequencing Preparation, Assembly, and Analysis

For each culture, 10 μg of high-quality (absorbance > 1.8 for both 260/230 and 260/280 spectrophotometric ratios), high-molecular weight DNA was used for SMRT sequencing on the Pacific Biosciences RSII platform. Each sample was loaded into a single-molecule-real-time (SMRT) cell that was prepped with the DNA Template Prep Kit 3.0 and DNA Polymerase Binding Kit P6 v2 (using the P6-C4 chemistry). Viral genomes were initially *de novo* assembled and polished using HGAP and Quiver for comparison to the PBCV-1 reference sequence in NCBI (Accession: NC_000852). Concomitantly, PacBio reads were aligned to the PBCV-1 reference genome to make epigenomic comparisons across biological replicates. Reference genome recruitment was done using the PacBio SMRT analysis platform (protocol version = 2.3.0, method = RS Resequencing). Instead of using the PacBio automated pipeline for DNA modification detection and motif analysis, we manually executed PacBio modification and motif detection tools from command-line to incorporate analysis options not offered in the GUI version. We used “ipdSummary.py” and “motifMaker.sh” to identify modifications and motifs, respectively. The command-line analysis produced four files for each sample that were collectively used for downstream analyses: “Modifications.gff,” “Modifications.csv,” “Motifs.gff,” and “Motif_Summary.csv.” We also repeated this process with different specified read mapping coverages in order to test for coverage effects, as the modification detection software is considered to be skewed toward false positive detection as sensitivity increases (i.e., high coverage). As we were primarily interested in m6A modification and wanted to decrease the chance of false positive detection, for the main body of results we present the data that was subsampled to 30X coverage as only 25X is reported sufficient for m6A identification at all genomic positions (PacBio, 2020). This was also appropriate because the DNA libraries were not prepared with Tet modification to support m5C analysis. Indeed, although PacBio documents report the capability to detect m5C that has not been Tet prepared (as long as coverage exceeds > 250x), the SMRT software does not actually provide an algorithm to support these analyses. All commands used for manual PacBio software analyses are described in Supplementary Methods.

### DNA Methylation Stability Analysis

A series of R functions were created to enable bioinformatic comparisons of methylation status between biological replicates. Using the function “all_motifs.R” the four output files generated from command-line modification and motif analysis were combined into one master file. This master file was cross-referenced with those generated from the biological replicates to determine stability of methylated bases. Specifically, we used PacBio’s “methylFrac” output to do this comparison, which reports how many reads aligning to a particular site are methylated with 95% confidence intervals. Thus, we calculated the average and standard deviation of site-specific methylFrac values between the three biological replicates to determine site-specific stability. Sites were grouped by methylation status (stably methylated; stably non-methylated; and stochastically methylated) and analyzed for commonalities in gene function and transcriptional information, where applicable. We also analyzed methylation status for GATC and CATG sequences on both their forward and reverse strands, since these sequences each reverse compliment with themselves. Thus, palindromes were defined and grouped as methylated on both strands, hemimethylated, or lacking methylation on both strands. Attempts to validate PacBio methylation patterns were done using restriction enzyme digests that target GATC tetramers in methylated or non-methylated forms.

## Results

### Viral Methyltransferases in the NCBI Database

To determine whether methyltransferase enrichment is common in “giant,” algal-infecting viruses, we queried the NCBI viral genome database for these enzymes (Supplementary Table S1). The results indicate that the top twenty eukaryotic virus hits were to algal-infecting viruses. Since these are in the “giant” class, we performed a correlation to define genome size as a driver of DNA methyltransferase enrichment, and found that this poorly explained the number of encoded DNA methyltransferases for eukaryotic viruses (*R*^2^ = 0.168). Rather, it appears that enzyme enrichment is driven by host-virus system types (i.e., eukaryotic phototrophs). This justifies further study of DNA methylation in chlorovirus PBCV-1 as a model system for understanding this DNA modification in eukaryotic, phototrophic host-virus systems.

### Distribution of m6A-Targeted Nucleotide Sequences in the PBCV-1 Genome

The DNA methyltransferase target motifs GATC and CATG occur a total of 3,498 times in the PBCV-1 genome. As palindromes, this represents 1,749 distinct genomic locations that could be methylated. In the PBCV-1 genome, which exhibits 40% GC content, GATC and CATG sequences would be randomly distributed once every 278 bp. In reality, GATC sites occur on average once every 388 bps, with an index of dispersion of 477, while CATG sites occur once every 364 bps with an index dispersion of 490. This contrasts with *E. coli* DNA sequences, which encode GATC sites once every 243 bp compared to a predicted frequency of every 256 bp (Barras and Marinus, 1988; Marinus and Lobner-Olesen, 2014). Efforts to identify PBCV-1 genomic regions enriched or depleted in these motif sites, again based on a context-independent window size of 278 bp, yields an association with many ORFs (Table 2 and Supplementary Table S2).

**TABLE 2:** Top ten PBCV-1 genomic regions enriched in GATC and/or CATG motifs using a sequence independent 278 bp window.

Location	Genes Impacted	ME	GATC	CATG	Txc	Annotations
315424–315838	*A656L*	4.5	9	0	Early	Collagen Triple Repeat (20 copies) [9.1E-11]
	*A658R*	4.5	9	0	Late	Hypothetical
111680–112010	*A219/222/226R*	4.5	4	5	Early	Glycosyltransferase [4.0E-6]
265596–265874	*A552R*	4.5	3	6	Early	Transcription Factor TFIID (TATA-binding) [3.1E-7]
244276–244690 A505L, a509R, a508R 4 6 2 Early Hypothetical protein	*A505L*	4	6	2	Early	Hypothetical protein
2772–3106	*A005R*	3.5	3	4	Early	Ankyrin repeat [6.3e-11]
167076–167422	*A330R*	3.5	3	4	Early-Late	Ankyrin repeat [1.3e-07]
108272–108602	*A214L*	3.5	4	3	Late	Hypothetical
286960–287258	*A596R*	3.5	1	6	Early-Late	Cytidine/Deoxycytidylate Deaminase [1.1E-26]
	*A598L*	3.5	1	6	Early-Late	Histidine Decarboxylase [3E-53
289432–289778	*A603aL*	3.5	1	6	Unknown	Hypothetical
	*A604L*	3.5	1	6	Early	Hypothetical
322108–322398	*A674R*	3.5	5	2	Early	Thymidylate Synthase [9E-57]

*Gene names denoted with an upper-case “A” are defined as major ORFs that have been detected in transcripts and/or proteomes, whereas minor ORFS have not been detected in those studies and are denoted with a lower-case “a” (Yanai-Balser et al., 2010; Dunigan et al., 2012). For clarity, we only show major ORFS here. Motif enrichment, denoted as ME, represents fold enrichment based on a window size of 278 base pairs (see section Materials and Methods). Locations spanning distances greater than 278 represent how far the window slid (4 bp increments) while still sharing the same motif sites. GATC and CATG columns list the number of times each motif was observed in the window. Txc denotes the stage at which transcripts for major ORFs are detected. E-values representing the highest confidence annotation derive from COG, Pfam, or KEGG hits.*

A brief glance at the top 10 enriched or depleted regions reveals some trends. First, it appears that early transcriptional genes are preferentially enriched in one or both of these motifs, though correlational analysis between transcriptional status of all ORFs (size normalized) and the amount of methylation revealed no relationship (*R* = 0.008 for GATC; *R* = 0.004 for CATG). Gene functions associated with these enriched regions include a variety of functions including regulatory elements, putative protein modifiers, and genes involved in thymidine production. On the other hand, there are several regions of the genome depleted in both tetramers. Almost all of the top ten most depleted regions are larger than 2kb, including the polycistronic region encoding eleven viral tRNAs.

**FIGURE 1:**
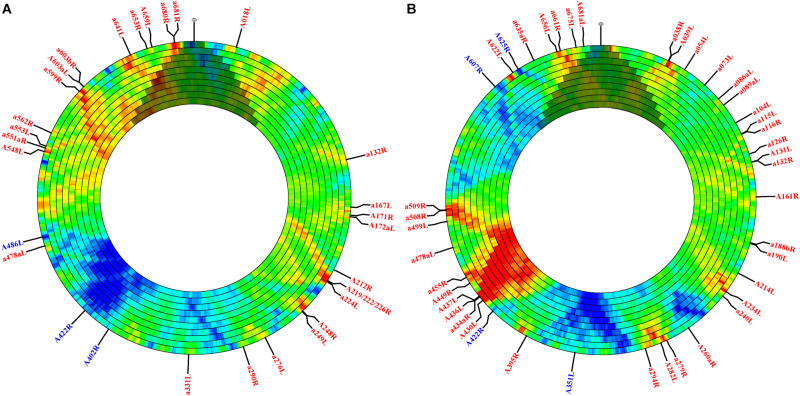
PBCV-1 motif enrichment and depletion determined in the context of local codon flexibility at different genomic increments. The PBCV-1 genome was analyzed for motif frequency of CATG **(A)** and GATC **(B)** tetramers. Panels show increasing window sizes, ranging from 4 to 40 kb moving from the outer to inner ring. Each ring increases incrementally by 4 kb, but the step size is always 4 kb. The Z-score is represented with color; red indicates more enriched scores and blue indicates more depleted scores. Green represents the expected frequency with no depletion or enrichment. Since the DistAMo software was created for circular chromosomes, there is artificial data coded across the termini (at 12 o’clock) for the linear PBCV-1 genome. This region has been shaded over to discourage faulty interpretation. Genes with significant Z-scores (greater than the absolute value of 2) are annotated by color (blue is enriched and red is depleted) and are included in Supplementary Table S2. Finally, the polycistronic region containing the viral tRNAs are not annotated in the DistAMo graphs since they are not annotated as genes at NCBI, though they are known to lie at the center of the genome between annotated genes *A326L* and *a331L.*

Although sequence-independent approaches have been widely used to estimate motif frequency, motif presence may be strongly governed by protein coding requirements. To assess motif frequency independent of this selective force, we used the open-source, online software DistAMo (Sobetzko et al., 2016). A broad analysis of all genes annotated with significant Z-scores, the metric for enrichment or depletion, indicated several things. First, although Z-scores are not quantitatively comparable to the frequency scores calculated in the sequence independent approach, many sites that were enriched or depleted according to the sequence independent approach were recapitulated using DistAMo (Figures 1A,B annotations; Supplementary Table S3). For example, the 278 bp region impacting gene *A219/222/226R* contained 4.5x more target sites than expected (with nearly equal representation of each type) according to the sequence independent approach. Results from DistAMo, however, indicated that only CATG sequences were enriched whereas GATC sites were dictated by coding requirements (Figure 1A annotations; Supplementary Table S3). DistAMo also confirmed that gene *A656L* was enriched in GATC sequences independent of protein coding requirements (Figure 1B). The DistAMo algorithm also indicated that minor, non-coding ORFs are more commonly enriched in methylation motifs (Figure 1 annotations; Supplementary Table S3), rather than the major ORFs. Indeed, only six instances of gene depletion were noted, and all of these represent major, protein-coding ORFs. This includes *A351L, A402R, A422R, A486L, A607R, and A625R*. Half of these (*A402R, A486L, A607R*) encode hypothetical proteins with no known homologs, but the remaining genes all encode orthologs for genome integration. *A351L* and *A422R* encode domains used by homing endonucleases for DNA binding, whereas *A625R* is a putative transposase ortholog. Last, since DistAMo can compute motif frequency at any defined window size, we expanded enrichment/depletion analysis to scales greater than genes (4–40 kb). At this scale, CATG and GATC sequence frequency counteracts one another in some regions, while there is an enrichment or depletion of both tetramers in other areas, such as consistent depletion in the middle of the genome (∼165 kb) (Figures 1A,B). Coincidentally, this region contains a polycistronic operon encoding 11 viral tRNA genes. It is only at the larger window size analysis that this region registers with an overall depletion in either methylation target.

### SMRT Sequencing of the PBCV-1 Genome

Past sequencing efforts indicate that the PBCV-1 genome has a length of 330,611 bp and a linear architecture with terminal inverted repeats that fold together to form hairpin loops (Dunigan et al., 2012). *De novo* assemblies generated in this study were 362,016 ± 3,579 bp with extra length discrepancies represented by duplicated inversions at each terminus (Supplementary Figure S2). A closer inspection of these sequences indicated the extra length represented sequence and/or assembler artifacts, an issue previously encountered during PacBio sequencing of another chlorovirus with this genomic architecture (Quispe et al., 2017). This was supported with BLAST homology searches of PBCV-1 ORFs annotated in the reference genome against the *de novo* assemblies, in which genes located in the terminal ∼2.5 kb of each end were duplicated. The corrected viral genome length yielded the expected genome size of ∼331 kb, thus validating a genome recruitment approach for methylome analyses.

**FIGURE 2:**
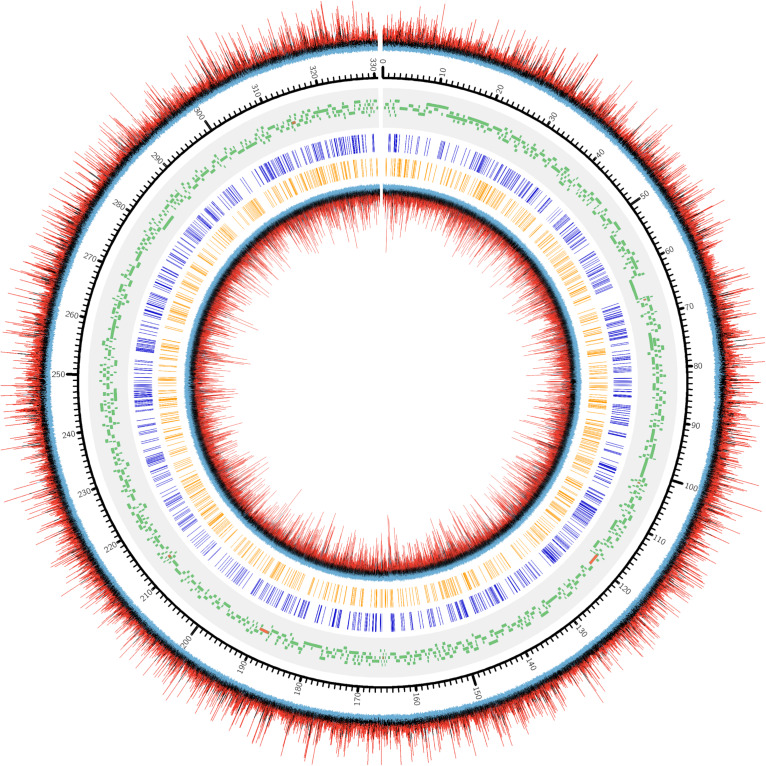
DNA modification induced kinetic events encountered during SMRT sequencing of one PBCV1 replicate (PBCV1-1C, Supplementary Figure S1). Starting from the outermost ring and going inward: (1) ipdRatio for each nucleotide on the forward strand, (2) genomic positions for PBCV-1 (kbp), 3) PBCV-1 potential protein coding sequences and tRNA encoding sequences (Dunigan et al., 2012), (4) CATG sites, (5) GATC sites, and (6) ipdRatio for each nucleotide on the reverse strand. The color coding of the ipdRatio is artificial to denote peak values, with a red peak denoting a value > 2. Black peaks indicate nucleotide kinetics similar to a non-modified base (ipdRatio ∼1), and blue peaks indicate a value < 0.5. This plot was made in CIRCOS (Krzywinski et al., 2009) with the intent of displaying ipdRatio peak height occurrence and diversity. The sequenced replicates exhibited CIRCOS plots with nearly imperceptible differences, which is why only one representative is shown here.

Genome recruitments accumulated 1466 ± 149 read coverage per nucleotide site per strand across the three biological replicates, with decreasing coverage occurring at the terminal ends (Supplementary Figure S3). Since high coverage is associated with an increased rate of false positive discovery (Feng et al., 2013), we decided to randomly sub-sample from the read pool to obtain a maximum recruitment coverage of 30-fold, which is appropriate for m6A detection. Using these settings, PacBio software identified a large number of nucleotides associated with modification, as indicated by a high interpulse duration ratio (ipdRatio), the primary metric for modification detection (Figure 2).

**FIGURE 3:**
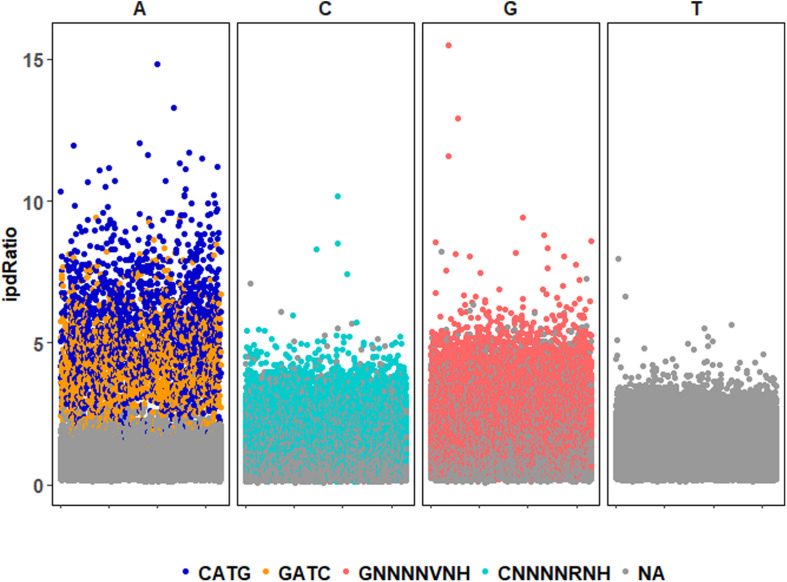
ipdRatio score for nucleotides in one replicate of the PBCV-1 genome at 30-fold read recruitment coverage. Dot color denotes association with a motif detected by motifMaker.sh. There are nearly imperceptible differences between the three replicates, which is why only one is shown here. The x-axis indicates genome position of each motif sequence.

72,170 nucleotide sites are marked with an ipdRatio > 2, which accounts for 10.9% of the PBCV-1 genome (including both forward and reverse strands). This represents ∼20.6x more sites than all of the available adenines associated with GATC and CATG sequences. While some of these events can putatively be accounted for by considering some to be secondary peaks known to form in close proximity to methylated sites, there is typically only one secondary peak per m6A site (Flusberg et al., 2010). Accounting for putative m6A background noise, as well as even the 1.86% of cytosines reported to be methylated from past studies, still yields ∼62,500 unaccounted nucleotide sites with an ipdRatio > 2. Some of these peaks occur in unexpected regions, including the 3,617 bp sequence that does not contain GATC or CATG sequences as determined from *in silico* analyses (starting at ∼62,000 base-pairs). A different visualization of this data in the context of motifs detected demonstrates that many cytosines, guanines, and thymines have high ipdRatio values, but only adenines separate as populations of modified motifs and non-modified adenines (Figure 3 and Supplementary Figure S4). Longer degenerate cytosine and guanine decorated motifs are detected by PacBio in each virus replicate, but at low frequency. Moreover, these are replaced with other low-frequency degenerate sequences at higher coverage analyses (Supplementary Table S4). Because these long degenerate strings are likely false positives (as PacBio analyses are skewed toward this; Biosciences, 2014) our analyses henceforward focus on the methylation status of the 3,498 adenines targeted by PBCV-1 DNA methyltransferases in GATC and CATG sequences.

To statistically distinguish modified and non-modified nucleotides, PacBio uses a Phred quality score (annotated as the ModificationQV). The software uses a default ModificationQV threshold of 30, corresponding to a *p*-value of 0.001, with higher QV values indicating modification. This scoring system allowed for better separation between modified and non-modified adenines (Supplementary Figures S4, S5), and defines 96.4 ± 0.3% and 84.1 ± 0.2% of CATG and GATC sites, respectively, as methylated (Supplementary Table S4). This accounts for 1.59 ± 0.004% of the total adenine pool, which is congruent with historic measurements of 1.45% (Van Etten et al., 1985), though it represents closer to ∼3200 as opposed to ∼2900 methylated targets. It also means that only 300 instead of a previously predicted 600 target sites are not methylated, though it is not clear if these sites are consistent between biological replicate populations. To determine site-specific methylation stability, we compared methylation status for each target site across three biological replicates.

**FIGURE 4:**
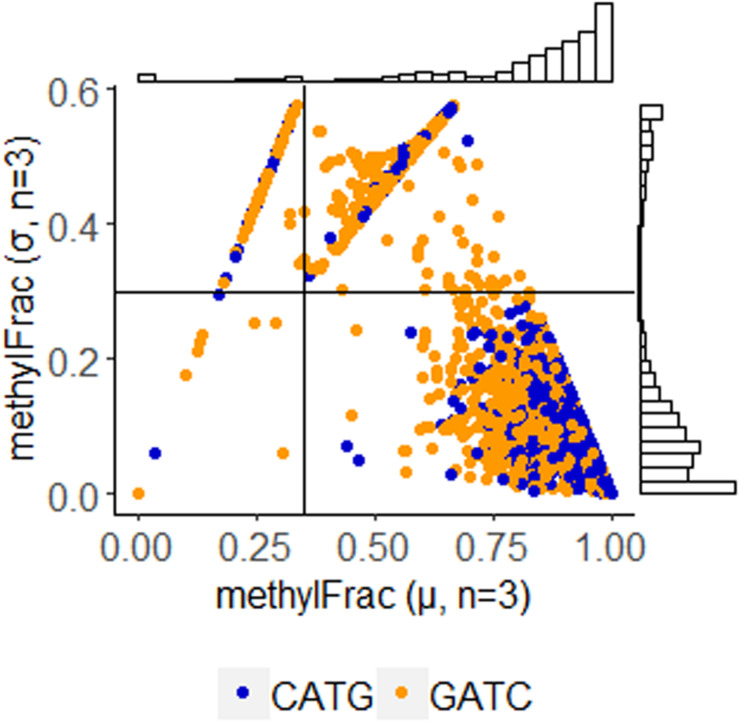
Methylation stability of GATC and CATG tetramers among PBCV-1 replicates, analyzed at 30-fold read recruitment coverage. Each dot represents the average number of reads that were methylated (x-axis) and the deviation between three biological replicates (y-axis). An average value approaching one indicates stable methylation, meaning the site was methylated in 100% of the 30 reads. Position along the y-axis indicates stability of the methylation status, as a value of zero indicates the methylation status is consistent between the three replicates. Coordinates were used to easily bin methylation status and frequency as stably non-methylated (Q1 – 73 events, 21.9% CATG, 57% GATC); complete methylation in only one of the three replicates (Q2 – 457 events, 23.1% CATG, 76.9% GATC), complete methylation in only two of the three replicates (Q3 – 143 events, 24.3% CATG, 75.7% GATC), and stable complete methylation in all replicates (Q4 – 2,825 events, 58.1% CATG, 41.9% GATC). Histogram plots provide an estimate of how many events occur with the given coordinates.

Using annotations directly made by PacBio software, we parsed sites identified with m6A modification. Stability of these sites was determined by comparison of methylFrac values, which represents the percent of reads aligning to that target site that have m6A modification. Mean and standard deviation calculation of this value between the three replicates enables a direct binning strategy for one of three modification characteristics: stably non-methylated sites (average methylFrac ∼ 0), variably methylated sites (methylFrac ∼ 1.0 in one or two of the three sequenced replicates, resulting in an average value of ∼0.33 or ∼0.66), and stably methylated sites (average methylFrac ∼1.0). By comparing methylFrac values averaged for each GATC and CATG site (treating targets on each strand as independent), it becomes clear that each characteristic type is represented (Figure 4). An estimate on the size of each population yields 2,825 tetramers (80.7%) that are almost always methylated, 457 tetramers (13.1%) that are methylated in two replicates, 143 tetramers (4.1%) that are methylated in only one replicate, and 73 tetramers (2.1%) that are almost never methylated. Thus, over 17% of target sites are variably methylated while the remaining 83% maintain a stable modification status.

**FIGURE 5:**
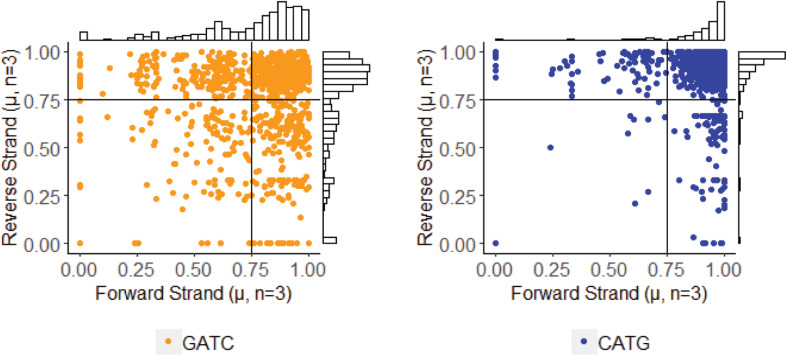
Methylation status of GATC and CATG palindromes across three sequenced replicates of PBCV-1. The status for the forward and reverse strand of a single palindrome are plotted for the separated motifs without variation information. Histogram plots provide an estimate of how many events occur with the given coordinates, with lines marking thresholds for defining complete methylation, hemimethylation, and stochastic methylation. A threshold of methylation in 75% of reads is typically used by PacBio for positive identification of methylation.

After determining methylation stability of each GATC and CATG site, we analyzed these within their palindromic context. Palindromic reverse complimentary sequences targeted by DNA methyltransferases can exist in one of three states: methylation occurring on both strands, methylation missing on both strands, or methylation occurring on only one strand (i.e., hemi-methylation). By mapping methylFrac status of adenines on both the forward and reverse strand of each palindrome, it is clear that all three types of palindromes are represented in the PBCV-1 genome, though CATG targets are more often fully methylated than GATC sequences (Figure 5). Among all 1,749 palindromes, 1,083 sites (61.9%) were methylated on average in > 75% of reads for both strands in a palindrome. Alternatively, 542 sites (31%) were defined as hemi-methylated with only one strand methylated in > 75% of reads. The remaining 124 sites (7.1%) were defined as more stochastic with methylation occurring on < 75% of reads on either strand.

Only three palindromes were annotated as stably not methylated in any reads mapping to either strand. This includes two loci located within neighboring genes encoding minor capsid proteins (CATG – *A383R*; GATC – *A384dL*) and one within a hypothetical protein (GATC – *A432R*). The genes impacted by these sites are all expressed late in the PBCV-1 infection cycle, their protein products are present in the PBCV-1 virion (Yanai-Balser et al., 2010; Dunigan et al., 2012), and they have no known homologs in the NCBI database outside of close viral relatives. All six target adenines of these palindromes had a ModificationQV value < 30, and all but one had an ipdRatio value < 2 (1.64 ± 0.78), supporting non-modification status. Confirmation of modification vacancies at these sites was attempted with digestion of the PBCV-1 genome using commercial restriction endonucleases *Dpn*I, *Dpn*II, and *Sau*3AI, which cut methylated GATC sites, non-methylated GATC sites, and GATC sites independent of methylation status, respectively (Supplementary Figure S6). Although *Dpn*II treatment yields a smear distinct from control DNA not treated with an enzyme, an expected ∼24,000 base-pair band representing the DNA between the two GATC non-methylated palindromes was absent. This suggests that some factor is inhibiting enzymatic digestion at the three locations marked by PacBio software as non-methylated.

**FIGURE 6:**
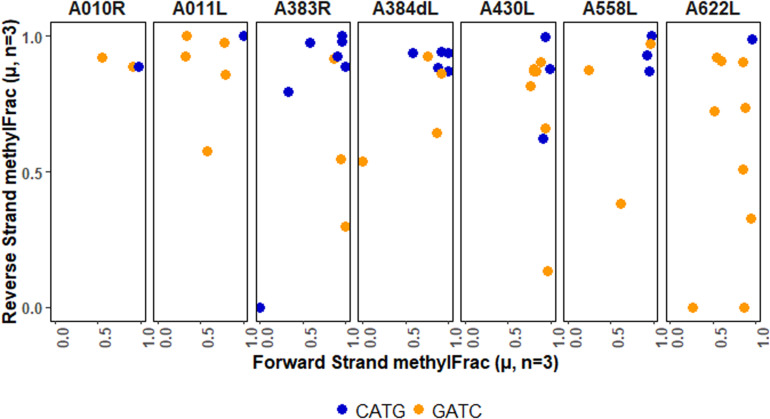
Methylation averages of reads aligning to the forward or reverse strand of palindromes falling within viral genes encoding capsid proteins. The major capsid protein is encoded by *A430L.*

Although the overall analysis of palindromes indicates that each type of methylation is present, functional implications may be better understood in the context of specific genomic units. There are several ways to do this, ranging from regulatory regions to whole genes. We chose to map palindrome methylation occurring in PBCV-1 genes that encode capsid proteins (Figure 6). In most cases methylation is stable, but there are a few completely non-methylated palindromes (*A383R*) or stable hemi-methylated palindromes (*A430L, A622L, A011L*). In general, though, there is typically a mix in the types of patterns observed per gene. These findings present opportunities to explore the consequence of this patterning on viral activity and fitness.

## Discussion

Many tools have been developed over the last few decades to allow molecular insight into host-virus interactions (Coy et al., 2018). Most notable are improvements in the “omics” techniques, enabling increasingly higher resolution studies ranging from single organisms to whole communities. Indeed, chlorovirus PBCV-1 has been subjected to genomics, transcriptomics, and proteomics, with the findings of this study establishing some of the first epigenomic stability observations in this system.

### Motif Enriched Regions Are Candidates for Investigating Regulation of Genes/Functions

*In silico* analyses indicate that GATC and CATG motif distribution in the PBCV-1 genome is not random. Sequence independent scoring demonstrated that the top ten enriched regions are associated with many major, protein-coding genes. Since 92.8% of the PBCV-1 genome is occupied by protein coding genes, this trend is somewhat expected. It is also congruent with methylation motif enrichment in prokaryotes whose genomes share a similar coding density of ∼90% (Land et al., 2015). On the other hand, DistAMo analysis focused on local codon redundancy indicated that most protein coding genes lose their enrichment status and minor, non-protein coding genes are instead enriched in GATC and/or CATG sequences. This seems more logical because minor genes are predicted to be non-protein coding and would thus not be under the selective pressure that DistAMo assumes. However, this is complicated by the fact that most minor genes overlap with protein coding genes, albeit as smaller units. It is also important to note that certain parts of a protein might be more flexible to amino acid substitutions than others, including regions not significantly impacting protein structure or function. Since DistAMo assumes amino acid sequence inflexibility across the entire length of a gene, it is difficult to conclude whether selection is greater on the protein sequence as opposed to subunits or intermediates of the gene (i.e., DNA or RNA secondary structure). In any case, genes/regions marked as enriched or depleted in both analyses provide higher confidence candidates that are truly enriched or depleted. For example, genes *A219/222/226R* and *A656L* might be good candidates for investigation into flexibility of the codon/amino acid sequence, as well as how variable methylation at these sites impacts the virus. It is especially interesting that methylation motif sites impacting *A656L* exist in a head-to-head orientation with *A658R*, and that these genes exhibit divergent transcriptional patterns. Perhaps methylation regulates which gene is transcribed early, while the other is transcribed late. Another similarity between PBCV-1 and bacteria is how motifs are dispersed. In *E. coli*, GATC motifs are never separated by more than 2 kb. This property has been hypothesized to promote mismatch repair efficiency, presumably because this function is less efficient when the GATC methyl-director is separated by greater distances (Marinus and Lobner-Olesen, 2014). Since PBCV-1 spacing is similar to *E. coli* (Table 2), and the *C. variabilis* NC64A host genome encodes a MutS homolog (XP_005846525), there might be a methyl-directed function associated with these two components. Although PBCV-1 does not encode its own MutS protein, several giant virus relatives encode their own homologs, suggesting MutS is important for virus fitness (Ogata et al., 2011). Finally, another observation from our sequence independent approach showed that GATC and CATG motifs counteract one another as enriched or depleted in regions analyzed at scales greater than genes (4–40 kb). Though it is not clear if this is meaningful for PBCV-1, it is interesting that GATC enrichment and depletion in *E. coli* marks the genome replication of origin and termination, respectively (Sobetzko et al., 2016). Thus, methylation might have similar implications for PBCV-1 chromosome maintenance.

### Motif Depleted Regions Are Candidates for Investigating Selection Against Regulation in Particular Genes/Functions

Another useful outcome of the *in silico* analysis is that it identified regions depleted in motifs targeted for methylation. The few genes marked as depleted by DistAMo represent all major, protein-coding ORFs, with about half having functions associated with genome integration. *A351L* and *A422R* encode domains used by homing endonucleases for DNA binding, whereas *A625R* is a transposase ortholog. That these proteins are all involved in genome integration processes might represent a negative selective pressure to side-effects of palindromes and/or methylation that can occur on these sites. In rice, hypermethylation occurs in transposable elements following whole genome duplication, a marker for angiosperm evolution (Zhang et al., 2015). Concomitantly, this modification inhibits transposition to stabilize the integrity of the rice chromosome and decrease nearby gene expression. Some of the larger chloroviruses encode many putative transposases (some with internal resolvases) and homing endonucleases (Fitzgerald et al., 2007), which are thought to be involved in genomic rearrangements and gene duplications (Jeanniard et al., 2013). These larger viruses are also sometimes more heavily methylated (Van Etten et al., 1991; Jeanniard et al., 2013), which might serve as a stabilizing force for chlorovirus genomes. An analysis of transposase methylation frequency across chloroviruses experiencing gene duplications might delineate this possibility. Another striking case of motif depletion in PBCV-1, which was identified by both *in silico* analysis approaches, was that the middle of the genome, where the viral tRNA polycistron is located, comprises one of the largest regions lacking either target motif. Palindrome depletion in tRNA, and even rRNA genes, has also been observed in bacterial genomes; these genes exhibit the lowest frequency of GATC motifs in *E. coli* (Marinus and Lobner-Olesen, 2014). Hypotheses related to this depletion in bacterial genes have suggested that selection occurs against palindromes to prevent secondary structure formation that might interfere with constitutive expression of these genes (Marinus and Lobner-Olesen, 2014). Though not related to methylation, this is worth considering.

### In-Complete Methylation of GATC and CATG Has Biological Consequences

Results from the PacBio software suggest PBCV-1 exhibits high, but not complete, methylation of GATC and CATG tetramers. Total adenine methylation accounts for 1.59 ± 0.004% of all adenines, which is close to historical HPLC measurements of 1.45% (Van Etten et al., 1985, 1991). It is also clear that GATC sites comprise the majority of non-methylated target sequences, whereas CATG sequences are completely methylated in most cases. That stochastic methylation occurs at a higher rate in GATC sites suggests this is not an artifact of the sequencing software, and is indeed a true biological phenomenon. Moreover, GATC sequences that are targeted for DNA methylation have been associated with a wide variety of confirmed and putative regulatory processes in other organisms (Casadesus and Low, 2006; Wion and Casadesus, 2006).

We identified three putative, completely non-methylated palindromes, but failed to confirm these with restriction digestion. Providing these are truly lacking methylation, there are some intriguing biological consequences at stake. Both of the adenine decorating methyltransferases in PBCV-1 are associated with RM systems. These RM systems have been shown to digest and recycle the host genome for viral DNA replication, while protecting the viral genome against self-digestion (Agarkova et al., 2006). Thus, there are potential deleterious consequences for PBCV-1 if a target site is not methylated. Indeed, this selective pressure is apparently strong enough to dictate complete methylation of RM targeted motifs in > 100 bacterial chromosomes analyzed with PacBio sequencing (Blow et al., 2016). Complete methylation would seem especially necessary for PBCV-1 since the viral restriction endonucleases are packaged in the virus particle (Agarkova et al., 2006). Failure to cleave the viral genome during *in vitro* restriction digestion indicates that the three palindromes identified by PacBio as fully non-methylated resist restriction by one of several means. First, it is possible that these sites are false negatives for m6A. This seems unlikely, given the kinetic fingerprints of these sites do not generally exhibit a high ipdRatio value on either strand that is indicative of m6A. Second, it is possible that some other type of modification is present on or at least in the vicinity of these palindromes, which prevents endonuclease recognition. If this were true, one would expect that this would interfere with polymerase kinetics to yield a high ipdRatio. That said, it is clear that some modifications do not elicit strong effects and are consequently poorly detected, if at all. Unlike m6A, native 5 mC protrudes into the major groove of DNA and is not involved in base-pairing, which is putatively why this modification elicits a more subtle impact on polymerase kinetics. Indeed, this is why Tet1 modification is used to improve the signal of 5 mC (Clark et al., 2013). Thus, there is no evidence to refute alternative modifications, though there is also not enough evidence to conclude that other modifications are present. At the same time, it is possible that these are false negatives deriving from poor *in silico* predictions of ipdRatio values for the non-modified control sequence. However, the original description of the *in silico* control demonstrated that it is effective at identifying all true positives at coverages similar to those used here (Feng et al., 2013). Thus, future investigation is needed to confirm the modification status of these three palindromes.

### Pacbio Sequencing Suggests PBCV-1 Has DNA Modifications Other Than Methylation

In considering ipdRatio values as the purported primary metric for modification (Flusberg et al., 2010; Blow et al., 2016), it is worthwhile to reiterate that several high ipdRatio events occur in the PBCV-1 genome that are not associated with motifs known to be targeted for methylation. To check if this is common in other systems, we analyzed ipdRatio distributions for *Escherichia coli* K-12 (MG1655) using data produced by and available from PacBio. This bacterium encodes three active DNA methyltransferases that recognize GATC, CCWGG, and AACNNNNNNGTGC/GCACNNNNNNGTT contexts. PacBio data indicated that > 99% of these sites were marked as methylated, which accounts for roughly 63,522 target sites. Total number of sites exceeding an ipdRatio > 2 yielded 154,230 sites, accounting for 1.7% of the bacterial genome (both forward and reverse strands). This represents an enrichment of only ∼2.4x in comparison to the ∼20.5x enrichment observed in PBCV-1. One might propose that the ∼65,000 nucleotides indicated as modified in PBCV-1 (not with methylation or a repeated, detectable motif) are an artifact of PacBio’s high error rate, which is ∼11–15% on average (Rhoads and Au, 2015). However, since the mapped reads were randomly sampled to 30-fold coverage, and the errors are random, this error rate reduces to <1%, which is far below the number of loci with high ipdRatio values.

Barring a poor *in silico* prediction of non-modified polymerase kinetics, it is possible that some other type of modification is responsible for the high number of peaks observed in PBCV-1. Indeed, this possibility has been hypothesized to explain thousands of unaccounted kinetic variation events that were observed in *E. coli* (Feng et al., 2013). This might be a promiscuous modification that is difficult to detect based on the current model of sequence frequency affiliation. One possible explanation is that oxidative stress induced lesions are occurring; these randomly occur in at least 20 different DNA base configurations (Cooke et al., 2003), and have been shown to elicit kinetic effects across multiple neighboring sites (Clark et al., 2011). That said, an algorithm to test this possibility does not currently exist in the PacBio software. In any case, oxidative stress is a considerable challenge for virus replication, which is presumably why so many giant viruses, including PBCV-1 (Kang et al., 2014), encode machinery to mitigate this stress (Wilhelm et al., 2017). Stress induced DNA modifications might become rampant in lytic virus progeny, thus representing a potential cause of some progeny being non-infectious. Indeed, chlorovirus PBCV-1 has a burst size of ∼1000 progeny, yet, only ∼30% of this population is capable of forming plaques (Van Etten et al., 1983, 1991). However, it should be noted that PBCV-1 also encodes a functional DNA repair enzyme (Furuta et al., 1997).

It is unlikely that cellular organisms, for which most modification analyses have been based on, would have as many oxidative stress induced DNA modifications as viruses because these organisms can repair these lesions as long as they are alive and growing under optimal culturing conditions. Viruses, on the other hand, might encounter an environment less conducive to DNA repair followed by genome packaging in a metabolically inactive virion. Moreover, our extraction protocol does not selectively acquire nucleic acid from only infectious virus. While we favor this idea, we also recognize the possibility that some type of modification not yet characterized, which has nothing to do with oxidative stress, could be responsible for some of the polymerase kinetics not caused by methylation. Recent computational studies have identified a variety of DNA modification systems (Iyer et al., 2013), and other PacBio work has confirmed the presence of novel modifications including phosphorothionation (Ahlgren et al., 2017). Giant viruses, whose predicted proteins are dominated by those with unknown functions might also be capable of novel DNA biomodifications. We stress this possibility in light of the fact that past HPLC-MS measurements of PBCV-1 nucleosides determined percent of methylated nucleotides based on relative pools as opposed to absolute quantitative measurements (Van Etten et al., 1985). This approach would mean that peaks associated with uniquely modified nucleotides, perhaps with drastically different retention times, would not be considered in nucleotide pool estimations. Moreover, enzymatic digestion of DNA to single nucleotides might disrupt some modifications, making HPLC incapable of detecting them.

### Hemi-Methylation Is Biologically Permissible for PBCV-1 Restriction Modification

A more common observation from this study is that hemimethylation can be common (∼31% of palindromes), though only a few of these sites are stable (2.51%). The status of these hemimethylated GATC sites is questionable given that their “non-methylated” strand often exhibits a large ipdRatio value. Despite that, it is not biologically impossible that hemi-methylation is a regular component of the PBCV-1 genome; this is known to occur in many cellular organisms including human cell lines (Ehrlich and Lacey, 2013; Xu and Corces, 2018) and bacteria (Fang et al., 2012). Moreover, the endonuclease targeting CATG sequences, M. *Cvi*AII, has been shown to not cleave hemi-methylated DNA (Luo et al., 2016). It is thus reasonable that the GATC targeting endonuclease is also unable to cleave hemimethylated targets, or to cleave targets at a much slower rate. This would be necessary considering viral replication time is much longer (a few hrs) than bacteria with stable methylation patterns (less than 20 min) (Fang et al., 2012). Altogether, these observations suggest hemimethylation is biologically permissible, and invites future investigation into whether this is a unique characteristic of these types of enzymes that relates to specific viral activity.

## Caveats and Conclusion

Finally, there are a few caveats to point out about our approach. First, we analyzed stability using the methylFrac value computed by PacBio software. Though this has been validated with external analyses (Beaulaurier et al., 2015), an inherent weakness is that the methylFrac analysis does not consider molecule specific effects. Namely, the 30 reads that align to a given site can derive from any number of molecules, each of which should in reality be considered as sub-populations or perhaps “quasi-species.” Other tools exist to analyze this data for population-level epigenomic variants (Beaulaurier et al., 2015), but our data could not be assessed this way as we used the *in silico* control data as opposed to sequencing a whole genome amplified control, which is currently a requirement for population-level analysis. Second, we sub-sampled our reads to a coverage of 30-fold, which is reportedly appropriate for methylation detection at all genomic positions according to PacBio. That said, the 95% confidence variables for the methylFrac value were at times quite large, which might be impacted by reads deriving from molecules with diverse methylation profiles. This effect cannot be accounted for until the SMALR package created by Beaulaurier et al. (2015) is updated to support analysis of read effects with an *in silico* control.

DNA methylation has been identified or inferred in many types of giant viruses using restriction mapping (Van Etten et al., 1991), cloning (Van Etten et al., 1985), and genomics (Fitzgerald et al., 2007; Schroeder et al., 2009; Moniruzzaman et al., 2014; Schvarcz and Steward, 2018; Stough et al., 2019). The enzymes responsible for these modifications are at times paired with a cognate restriction endonuclease or an endonuclease that only cleaves one strand of the dsDNA in a site-specific manner (nicking enzyme) (e.g., CCD; Xia et al., 1988) or R/AG (Zhang et al., 1998), thus forming a viral restriction modification system, as is the case for PBCV-1. Though giant virus RM-systems have been functionally confirmed in only the chloroviruses, bioinformatic analyses based on homology and gene co-localization suggest that RM-systems are also utilized by Chrysochromulina parva virus (Cpv-BQ2) (Stough et al., 2019). Nevertheless, in chloroviruses the majority (∼75%) of DNA methyltransferases are not paired with a site-specific endonuclease and are instead annotated as “orphan” DNA methyltransferases (Van Etten and Dunigan, 2012). Orphan DNA methyltransferases have been identified as regulators of cellular and viral activities in bacteria (Sternberg and Coulby, 1990; Murphy et al., 2013), though a function has not yet been proposed for them in giant viruses. These giant virus enzymes are not likely used for chlorovirus restriction evasion because there is no need to protect DNA from eukaryotic hosts, which do not encode restriction endonucleases. This observation, combined with the fact that chloroviruses and their giant virus relatives encode some of the largest numbers of putative DNA methyltransferases among viruses, suggests a novel and seemingly biologically important use of DNA methyltransferases for viral fitness. This is supported by the observation that phylogeny of some of these enzymes reflects a long evolutionary history within viruses, instead of a recent acquisition from cellular organisms by horizontal gene transfer. Complementing this concept, is that other enzymes encoded by the chloroviruses have seemed to adapt to side-effects of genomic methylation. For example, DNA topoisomerase II from PBCV-1 cleaves DNA 30-50x faster than human type II topoisomerases (Fortune et al., 2001). This rate is believed to stem from an adaptation to higher incidences of DNA methylation in chloroviruses (Dickey et al., 2005), as methylation is known to slow type II topoisomerase processing much like it does with DNA polymerase (Dickey et al., 2005). It would be interesting to see if different variations of palindrome methylation impact topoisomerase activity differently. It is also possible that these markers assist with genome condensation for viral DNA packaging (Wulfmeyer et al., 2012). In another example, hemimethylated palindromes have been shown to control promotor activation, in some cases allowing gene expression only transiently following DNA replication (Casadesus and Low, 2006). Though we did not see an obvious correlation between transcriptional profile and methylation here, it is possible that the methylation profile during an active viral infection might better explain transcriptional orchestration of PBCV-1. In any case, information provided here establishes a useful framework for investigating DNA methylation in chlorovirus PBCV-1, as well as initiating these studies in other systems with more “orphan” DNA methyltransferases.

## Data Availability Statement

The datasets generated for this study can be found in the Bioproject Database (Accession PRJNA546117) on the National Center for Biotechnology Information website.

## Author Contributions

SC and SW conceived the design of these experiments. EG created python code for some of the *in silico* analyses, while SP created and executed python code to identify viral DNA methyltransferases in public genome repositories. MH, NA, and JP conducted PacBio sequencing on their in-house platform and did *de novo* assembly. JV provided the source materials for study. SW provided funding. All other analyses, code, and data curation were completed by SC. All authors contributed to manuscript production and editing.

## Conflict of Interest

The authors declare that the research was conducted in the absence of any commercial or financial relationships that could be construed as a potential conflict of interest.

## Footnotes

1 https://www.genome.jp/virushostdb/
