# Supplementary material for: SMRT Sequencing of Paramecium Bursaria Chlorella Virus-1 Reveals Diverse Methylation Stability in Adenines Targeted by Restriction Modification Systems
Source: Front Microbiol. 2020 May 19;11:887. doi: 10.3389/fmicb.2020.00887 (PMC7248222; doi:10.3389/fmicb.2020.00887)
Supplement: Supplementary file 1 [file Presentation_1.pdf]

Supplemental Methods

Commands used for Modification and Motif Detection (ipdSummary.py; motifMaker.sh)

Paths to directories specifying files have been simplified for brevity. A full description of the arguments included here can be found from the help manual included in the command-line tool. We admit that some usage might need to be altered as the software has gone through updates since our analyses were completed. The four output files that are generated from this (in their final form) are highlighted in red. This process was reiterated to generate files at coverage (X) of 30, 95, 170, 255, and full coverage. It is also important to note that we maintained use of the default minScore threshold for identifying motifs based on a modificationQV score, though adjustment is recommended to prevent false positive motif identification. Typically, this minScore threshold can be chosen based on a break in the modificationQV values of nucleotides as a function of per-strand coverage; modified bases have a higher modificationQV value. However, choosing this threshold is slightly arbitrary in that some nucleotide sites cannot be confidently assigned (Supplemental Figure 5). Indeed, at the maximum coverage there is not a clear break to set a minScore threshold (data not shown).

```
$ ipdSummary.py /PBCV1-1C_AlignedReads.cmp.h5
--reference /PBCV_1.fasta
--gff /PBCV1-1C_Modifications.gff
--csv /PBCV1-1C_Modifications.csv
--identify m6A,m4C,m5C_TET
--methylFraction
--maxCoverage X
```

```
$ motifMaker.sh find
--fasta /PBCV_1.fasta
--gff /PBCV1-1C_Modifications.gff
--minScore 30.0
--output /PBCV1-1C_Motif_Summary.csv
```

```
$ motifMaker.sh reprocess
--fasta /PBCV_1.fasta
--gff /PBCV1-1C_Modifications.gff
--motifs /PBCV1-1C_Motif_summary.csv
--output /PBCV1-1C_Motifs.gff
```

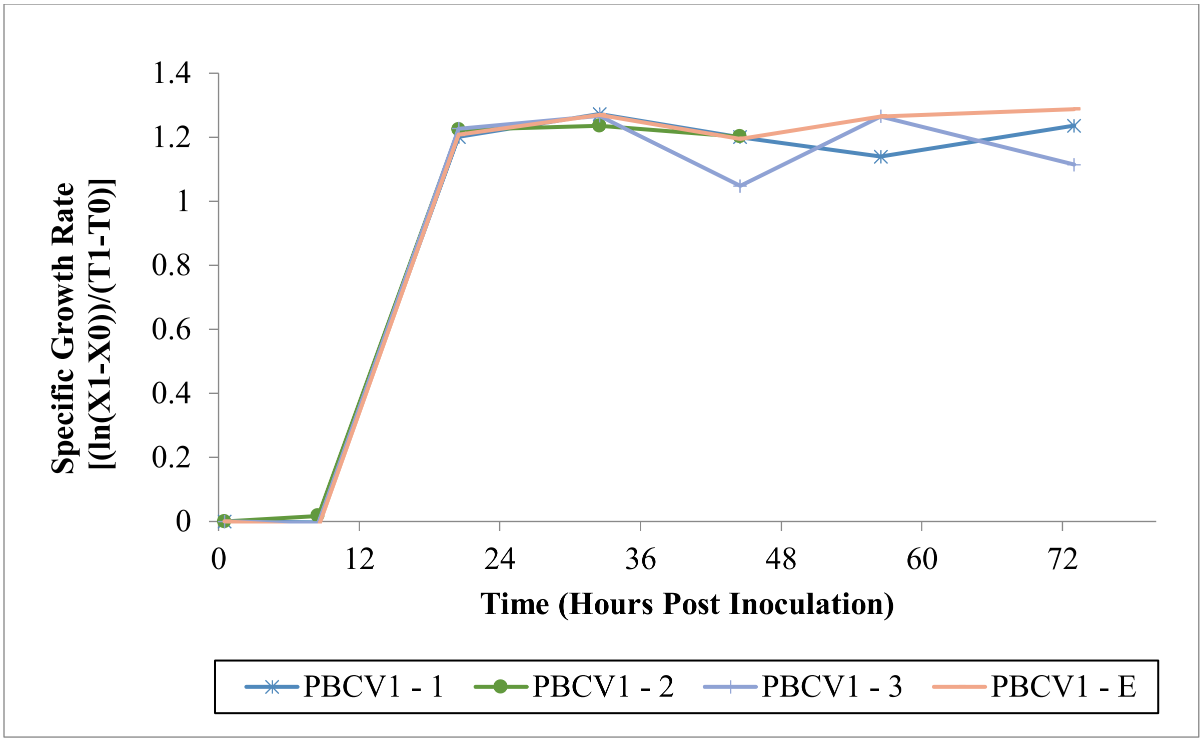

Figure S1. Growth dynamics of the host *C. variabilis* NC64A cultures prior to PBCV-1 infection at 72 h post-inoculation. Three of these cultures were used for Pacbio sequence, excluding PBCV1-3.

Table S1. Viruses encoding the most methyltransferase genes. Only the top twenty viral genomes of each host domain are displayed. (n= 10,708 virus genomes)

Host	ID	Virus Type	Viral Species/Strain	MTase	Genome (kbp)
Prokaryote	GCA_002593925	Cyanophage	Synechococcus phage ACG-2014f	5	23
Prokaryote	GCA_002593945	Cyanophage	Synechococcus phage ACG-2014f	5	22
Prokaryote	GCA_002594045	Cyanophage	Synechococcus phage ACG-2014f	5	22
Prokaryote	GCA_002594185	Cyanophage	Synechococcus phage ACG-2014f	5	22
Prokaryote	GCA_002594565	Cyanophage	Synechococcus phage ACG-2014f	5	23
Prokaryote	GCF_000898015	Bacteriophage	Cronobacter phage vB_CsaM_GAP32	5	36
Prokaryote	GCA_002593785	Cyanophage	Synechococcus phage ACG-2014f	4	23
Prokaryote	GCA_002593805	Cyanophage	Synechococcus phage ACG-2014f	4	23
Prokaryote	GCA_002593845	Cyanophage	Synechococcus phage ACG-2014f	4	23
Prokaryote	GCA_002593885	Cyanophage	Synechococcus phage ACG-2014f	4	22
Prokaryote	GCA_002593985	Cyanophage	Synechococcus phage ACG-2014f	4	22
Prokaryote	GCA_002594025	Cyanophage	Synechococcus phage ACG-2014f	4	22
Prokaryote	GCA_002594065	Cyanophage	Synechococcus phage ACG-2014f	4	22
Prokaryote	GCA_002594085	Cyanophage	Synechococcus phage ACG-2014f	4	22
Prokaryote	GCA_002594105	Cyanophage	Synechococcus phage ACG-2014f	4	22
Prokaryote	GCA_002594165	Cyanophage	Synechococcus phage ACG-2014f	4	22
Prokaryote	GCA_002594385	Cyanophage	Synechococcus phage ACG-2014f	4	23
Prokaryote	GCA_002594405	Cyanophage	Synechococcus phage ACG-2014f	4	23
Prokaryote	GCA_002594485	Cyanophage	Synechococcus phage ACG-2014f	4	23
Prokaryote	GCA_002594505	Cyanophage	Synechococcus phage ACG-2014f	4	22
Eukaryote	GCF_000873685	NCLDV	Chlorella virus NY2A	18	369
Eukaryote	GCF_000871245	NCLDV	Chlorella virus AR158	16	345
Eukaryote	JX997170	NCLDV	Chlorella virus IL-5-2s1	15	345
Eukaryote	JX997182	NCLDV	Chlorella virus NY-2B	15	345
Eukaryote	JX997172	NCLDV	Chlorella virus MA-1D	15	340
Eukaryote	JX997183	NCLDV	Chlorella virus NYs1	13	348
Eukaryote	JX997160	NCLDV	Chlorella virus CVB-1	11	319
Eukaryote	HQ704802	NCLDV	Organic Lake Phycodnavirus 1	8	345
Eukaryote	GCF_000922335	NCLDV	Aureococcus anophagefferens virus	6	371
Eukaryote	GCF_000847045	NCLDV	Chlorella virus PBCV-1	5	331
Eukaryote	GCF_000889395	NCLDV	Cafeteria roenbergensis virus BV-PW1	5	617
Eukaryote	GCF_000905435	NCLDV	Ostreococcus lucimarinus virus OlV5	5	186
Eukaryote	GCF_000907415	NCLDV	Phaeocystis globosa virus 16T	5	460
Eukaryote	JX997163	NCLDV	Chlorella virus CVM-1	5	327
Eukaryote	KY322437	NCLDV	Tetraselmis virus 1	5	668
Eukaryote	JX997176	NCLDV	Chlorella virus NE-JV-1	5	327
Eukaryote	GCF_000887855	NCLDV	Ostreococcus tauri virus 2	4	184
Eukaryote	GCF_001887825	NCLDV	Only Syngen Nebraska Virus 5	4	327
Eukaryote	JX997159	NCLDV	Chlorella virus CVA-1	4	327
Eukaryote	JX997154	NCLDV	Chlorella virus AP110A	4	327

Table S2. Top ten PBCV-1 genomic regions depleted in motifs using a sequence-independent 278 bp window

Location	Genes Impacted	MD	Txc	Annotations
62092 – 65709	*A121R*	-26	Early-Late	Hypothetical protein
	A122/123R		Early	Autotransporter adhesion [1.0E-12] (glycoprotein repeat)
10109-12557	*A014R*	-18	Late	Hypothetical protein
	A018L		Late	Glycoprotein repeat [1.2E-11]
	a016L, a017L			
126568-128978	*A251R*	-17	Early	M.CviAII (CATG) Methyltransferase
	A252R		Early	R.CviAII (CATG) Restriction Endonuclease
	A253R		Early	Hypothetical protein
	A254R		Late	Hypothetical protein
	a253aR, a252bL, a251bL, a252aL, a251aL			
180346-182661	*A363R, A368L*	-17	Early	Hypothetical proteins
	A366L		Early-Late	Hypothetical protein
	a367R, a365L			
299832-302126	*A623aL*	-17	n/a	Hypothetical protein
	A623L		Early	AN1-like Zinc finger [1.7E-12]
	A624R		Late	Predicted membrane protein [3.4E-26]
	A625R		Late	Transposase IS605 OrfB Family [2.0E-20]
	A627R		Late	Hypothetical protein
	a626L, a626aR			
297089-299232	*A619L, A620L, A621L*	-15	Late	Hypothetical proteins
	A622L		Late	Capsid Protein
	a621bL, a621aR, a620aR			

Gene names denoted with an upper-case 'A' are defined as major ORFs that have been detected in transcripts and/or proteomes, whereas minor ORFS have not been detected and are denoted with a lower-case 'a' (Yanai-Balser et al., 2010; Dunigan et al., 2012). Motif depletion, denoted as MD, represents fold depletion of motifs based on a window size of 278 base pairs (see Materials and Methods). Txc denotes the stage at which transcripts for major ORFs have been detected. Annotations are listed for only the major ORFs and tRNAs; only one is listed per gene, and those given with an e-value represent the highest confidence annotation based on COG, Pfam, or KEGG hits. Genes listed with n/a were either not detected in transcriptional studies, or are confirmed non-protein coding genes.

Table S2 (Continued)

Location	Genes Impacted	MD	Txc	Annotations
195248-197302	*A401R, A403R*	-15	Early-Late	Hypothetical proteins
	A402R, A404R, A405R		Late	Hypothetical proteins
	A404aL		n/a	Hypothetical protein
253391-255424	*A532L*	-15	Late	Hypothetical protein
	A532aL, A534R		n/a	Hypothetical proteins
	A533R, A535L, A536L		Early-Late	Hypothetical proteins
163902-165909	*A328L*	-14	n/a	Hypothetical protein
	A329R		Late	Hypothetical protein
	a329aL		n/a	
	Lys-3, Tyr-1, Ile-1, Leu-1, Lys-2, Asn-2, Arg-1, Lys-1, Asn-1, Pseudo-tRNA-1		n/a	9/10 Putatively functional tRNAs
39482-41467	*A075L*	-14	Early-Late	Exostosin Family [5.5E-9]
	A075cR		n/a	Hypothetical protein
	A075bl		n/a	Hypothetical protein
	A076L		n/a	Hypothetical protein
	A077L		Early	Hypothetical protein
	A078R		Early	N-carbamoylputrescine amidohydrolase
	a075aR			

Gene names denoted with an upper-case 'A' are defined as major ORFs that have been detected in transcripts and/or proteomes, whereas minor ORFS have not been detected and are denoted with a lower-case 'a' (Yanai-Balser et al., 2010; Dunigan et al., 2012). Motif concentration, denoted as MD, represents fold depletion of motifs based on a window size of 278 base pairs (see Materials and Methods). GATC and CATG columns list the number of times each motif was observed in the window. Txc denotes the stage at which transcripts for major ORFs have been detected. Annotations are listed for only the major ORFs and tRNAs; only one is listed per gene, and those given with an e-value represent the highest confidence annotation based on COG, Pfam, or KEGG hits. Genes listed with n/a were either not detected in transcriptional studies, or are confirmed non-protein coding genes.

Table S3. PBCV-1 ORFs enriched or depleted in GATC or CATG motifs

Gene Name	motif	zScore	Start	End	Accession
a478aL	GATC	4.2	231306	231812	NP_048835.2
a126R	GATC	3.87	66620	66820	NP_048474.1
a508R	GATC	3.75	244334	244567	NP_048864.1
a661R	GATC	3.65	316543	316851	NP_049017.1
A437L	GATC	3.64	212519	212830	NP_048794.2
a509R	GATC	3.57	244423	244728	NP_048865.1
a279R	GATC	3.54	142146	142364	NP_048633.1
a434aR	GATC	3.45	212284	212472	YP_004678953.1
a116R	GATC	3.4	59398	59643	NP_048464.1
a038R	GATC	3.35	23584	23823	NP_048386.1
a190L	GATC	3.19	97525	97743	NP_048537.1
A436L	GATC	3.04	212299	212490	NP_048793.2
a499L	GATC	3.04	240483	240716	NP_048855.1
A622L	GATC	3	298138	299700	NP_048978.1
a086aL	GATC	2.93	45101	45229	YP_004678889.1
a294R	GATC	2.88	149926	150150	NP_048648.1
A234L	GATC	2.85	115777	116103	NP_048582.1
A214L	GATC	2.67	108265	108672	NP_048561.1
a089aL	GATC	2.67	47826	47972	YP_004678892.1
a132R	GATC	2.6	69533	69805	NP_048480.1
a635aR	GATC	2.59	307064	307258	YP_004678992.1
a054L	GATC	2.58	29800	30123	NP_048402.1
a073L	GATC	2.55	38417	38626	NP_048421.1
A430L	GATC	2.47	210155	211468	NP_048787.1
a240L	GATC	2.46	117770	117967	NP_048588.1
A260aR	GATC	2.41	133700	133897	NP_048614.3
a675L	GATC	2.4	321966	322334	NP_049031.1
A282L	GATC	2.4	143630	145339	NP_048636.1
A656L	GATC	2.22	315127	315849	NP_049012.2
a115L	GATC	2.18	59265	59495	NP_048463.1
A449R	GATC	2.18	217799	218380	NP_048806.1
A039L	GATC	2.15	23623	24078	NP_048387.1
A161R	GATC	2.14	81345	81716	NP_048509.1
A681aL	GATC	2.11	324693	324869	YP_004678999.1
a104L	GATC	2.1	55054	55344	NP_048452.1
A395R	GATC	2.09	191505	191753	NP_048752.1
A131L	GATC	2.09	69359	69769	NP_048479.1

Table S3. (continued)

a188bR	GATC	2.08	97258	97398	YP_004678909.1
a455R	GATC	2.03	220218	220661	NP_048812.1
A607R	GATC	-2.04	290633	291808	NP_048963.2
A351L	GATC	-2.2	173636	174712	NP_048708.1
A422R	GATC	-2.21	205267	206259	NP_048779.2
A625R	GATC	-2.39	300424	301722	NP_048981.2
a553L	CATG	4.15	265624	265839	NP_048909.1
A172aL	CATG	3.38	88944	89111	YP_004678906.1
a132R	CATG	3.14	69533	69805	NP_048480.1
a167L	CATG	3.09	85677	85880	NP_048515.1
A603aL	CATG	2.84	289390	289575	YP_004678983.1
a478aL	CATG	2.8	231306	231812	NP_048835.2
a641L	CATG	2.8	308469	308726	NP_048997.1
a224L	CATG	2.79	112197	112463	NP_048572.1
a551aR	CATG	2.75	264946	265074	YP_004678972.1
a680R	CATG	2.68	323837	324100	NP_049036.1
A219/222/226R	CATG	2.56	110893	112926	NP_048569.4
a331L	CATG	2.49	167096	167299	NP_048687.1
a249L	CATG	2.42	125191	125499	NP_048598.1
A212R	CATG	2.31	107615	107782	NP_048559.2
a276L	CATG	2.31	140462	140746	NP_048630.1
a290R	CATG	2.27	148366	148773	NP_048644.1
a603bR	CATG	2.24	289445	289591	YP_004678984.1
a562R	CATG	2.17	270374	270571	NP_048918.1
a681R	CATG	2.14	323857	324081	NP_049037.1
A018L	CATG	2.14	12367	16374	NP_048366.1
a653R	CATG	2.08	314450	314647	NP_049009.1
a599R	CATG	2.07	287117	287656	NP_048955.1
A171R	CATG	2.05	87904	89067	NP_048519.1
A548L	CATG	2.02	263043	264530	NP_048904.2
A248R	CATG	2.01	124712	125638	NP_048597.1
A659L	CATG	2	316000	316578	NP_049015.2
A402R	CATG	-2.01	195325	196008	NP_048759.1
A486L	CATG	-2.2	234401	234859	NP_048842.1
A422R	CATG	-2.3	205267	206259	NP_048779.2

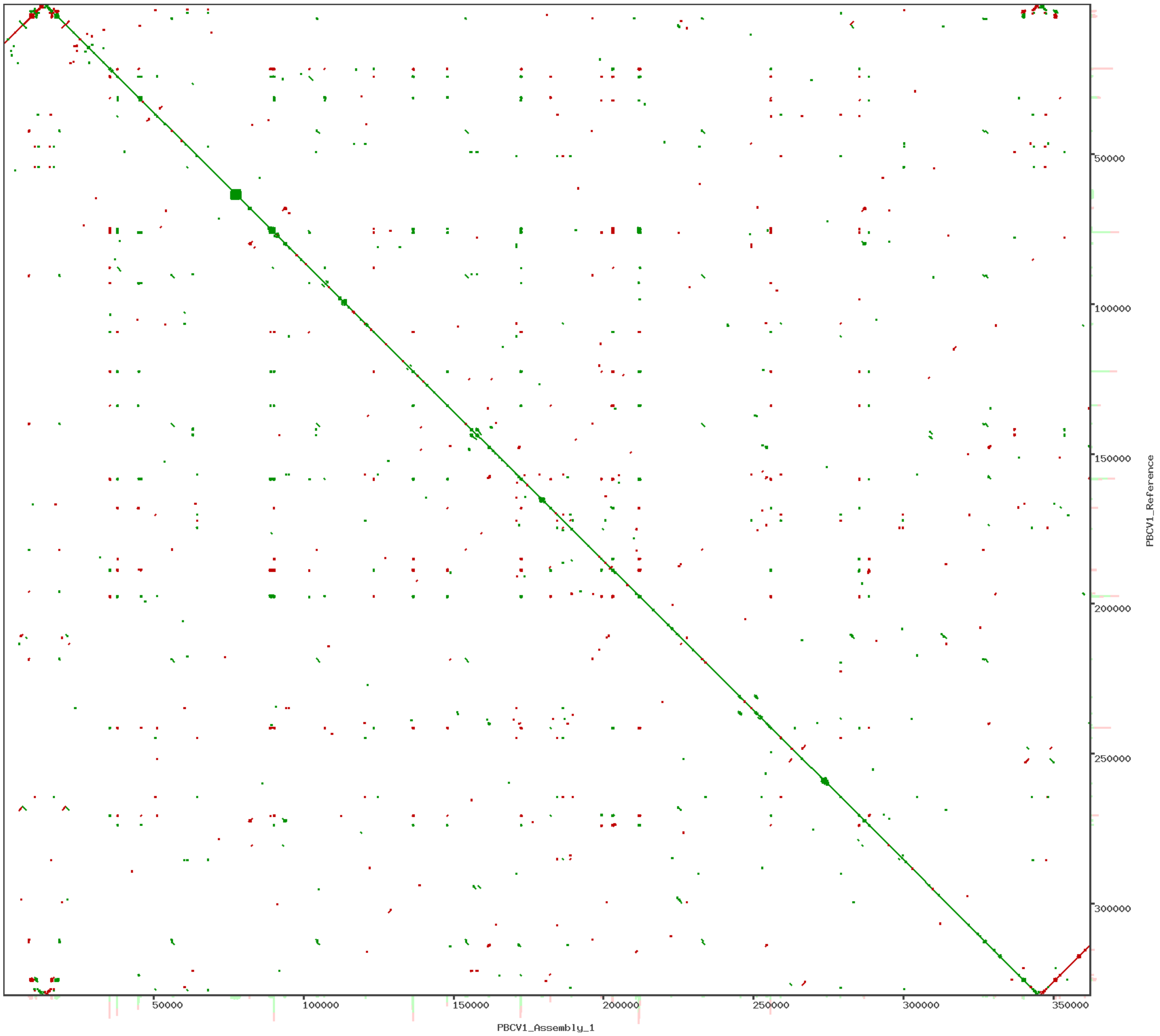

Figure S2A. Dot plot alignments of PBCV1_1C *de novo* assembly (x-axis) against the reference genome (y-axis).

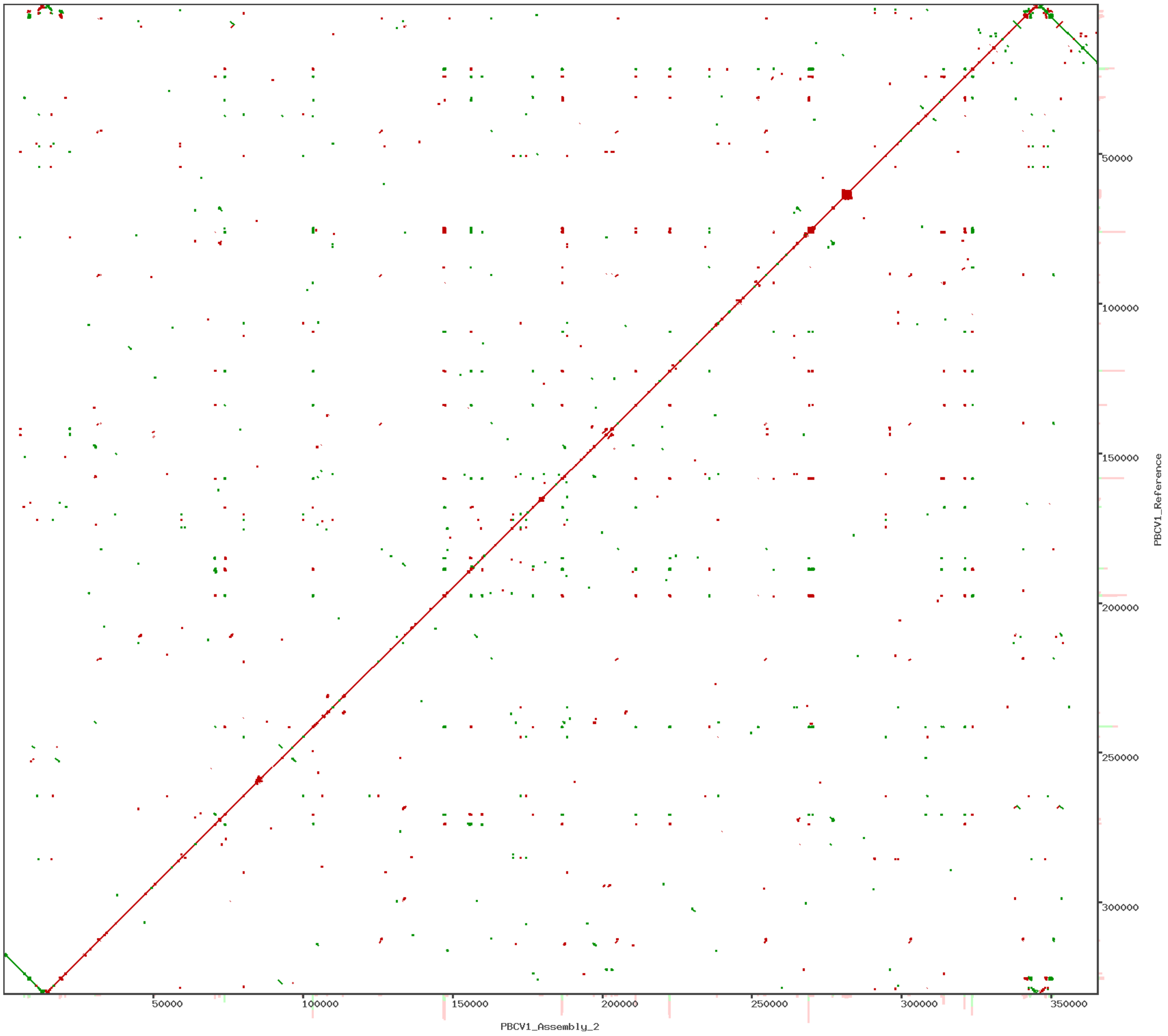

Figure S2B. Dot plot alignments of PBCV1_2A *de novo* assembly (x-axis) against the reference genome (y-axis).

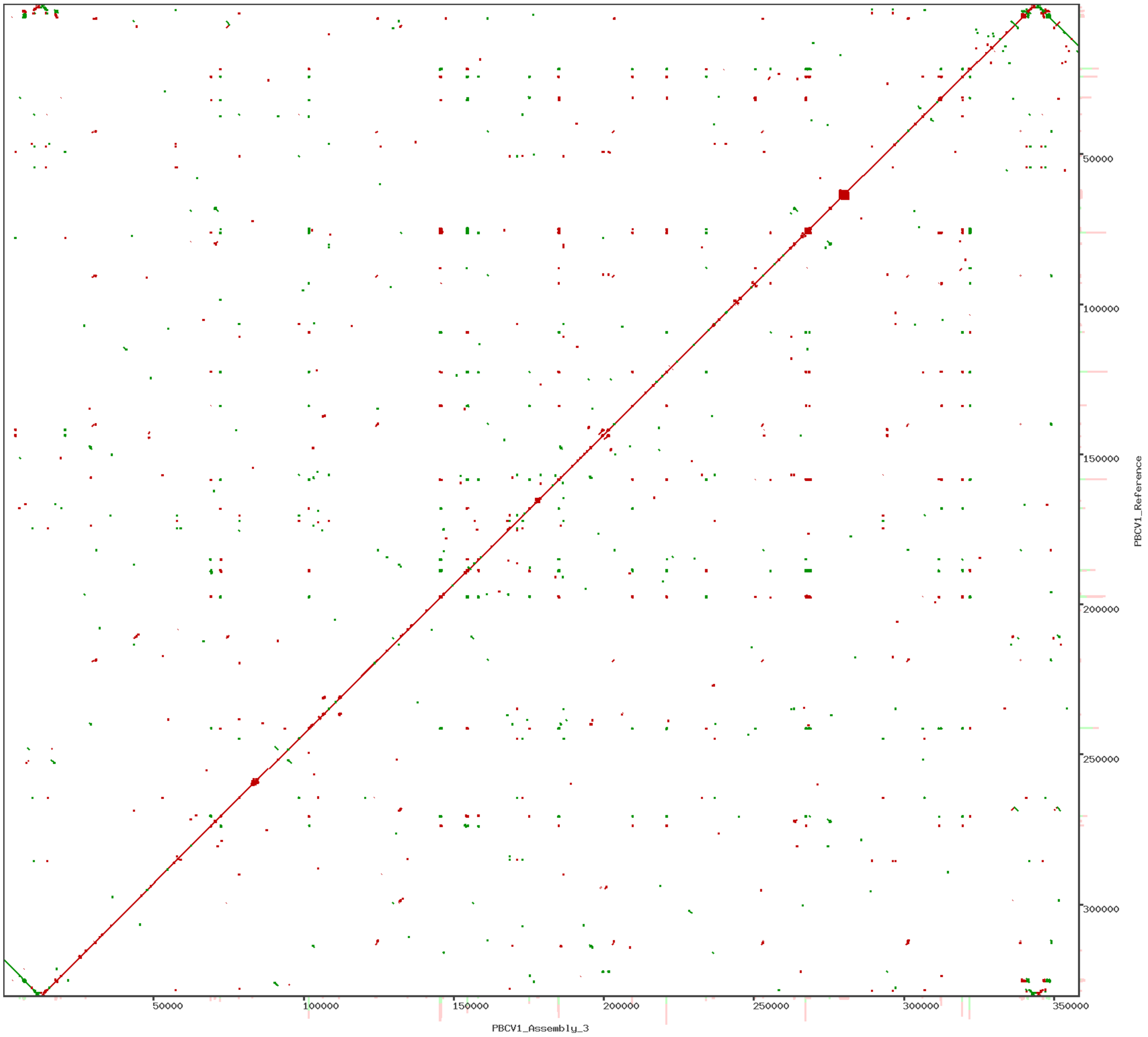

Figure S2C. Dot plot alignments of PBCV1_E1 *de novo* assembly (x-axis) against the reference genome (y-axis).

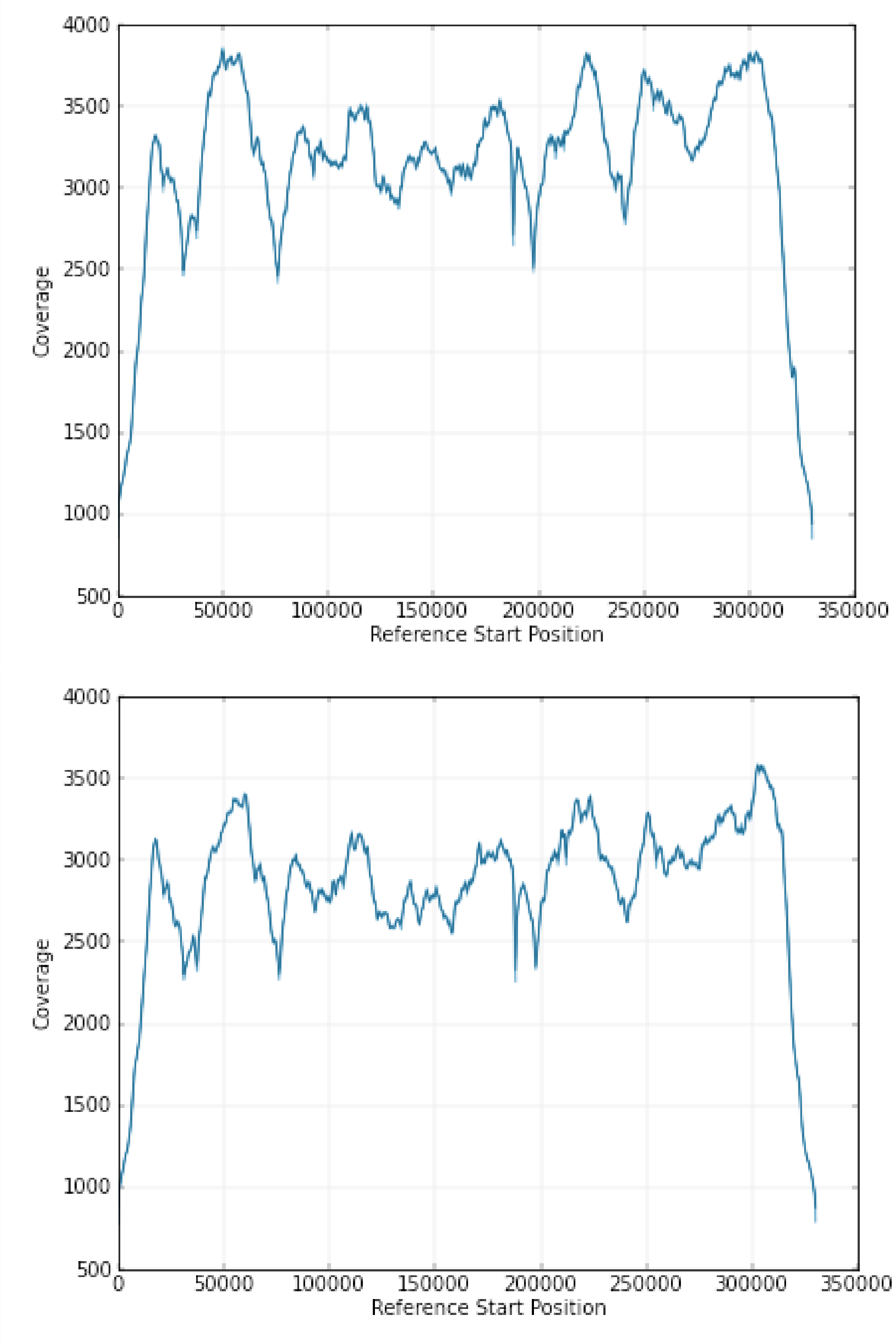

Figure S3. Pacbio read coverage based on recruitments for PBCV1_1C (top), PBCV1_E1 (bottom) to the PBCV-1 reference genome.

Table S4A. DNA sequences in the PBCV-1 genome identified as modified using Pacbio's DNA modification analysis tools. (Coverage: 30X)

Replicate	motifString	modificationType	fraction	nDetected	nGenome	meanScore	meanIpdRatio	objScore
PBCV1-1C	CATG	m6A	0.9672586	1743	1802	52.807804	5.4444346	89327.22
PBCV1-1C	GATC	m6A	0.8443396	1432	1696	50.525837	4.466902	62132.953
PBCV1-1C	GNNNNVNH	modified_base	0.052710593	3792	71940	35.31804	3.0786736	9474.864
PBCV1-1C	CNNNNRNH	m5C	0.019287998	1050	54438	37.418095	2.5839715	1124.6812
PBCV1-2A	CATG	m6A	0.9672586	1743	1802	52.807804	5.4444346	89327.22
PBCV1-2A	GATC	m6A	0.8443396	1432	1696	50.525837	4.466902	62132.953
PBCV1-2A	GNNNNVNH	modified_base	0.052710593	3792	71940	35.31804	3.0786736	9474.864
PBCV1-2A	CNNNNRNH	m5C	0.019287998	1050	54438	37.418095	2.5839715	1124.6812
PBCV1-E1	GNATWATNGCA	modified_base	1	5	5	38.6	2.722	193
PBCV1-E1	CATG	m6A	0.963929	1737	1802	52.84226	5.422868	88801.79
PBCV1-E1	GATC	m6A	0.8402123	1425	1696	50.331226	4.3777704	61320.055
PBCV1-E1	GNNNNVNH	modified_base	0.05321101	3828	71940	35.40935	3.1023834	9671.443
PBCV1-E1	GNVVNTBH	modified_base	0.04541603	536	11802	34.830223	2.9693081	1155.0726
PBCV1-E1	CNNNNRNH	m5C	0.021161688	1152	54438	37.25434	2.5690968	1335.4408

Note: Pacbio advertises 25X as sufficient coverage to detect all real m6A modifications in a sample sequence

Table S4B. DNA sequences in the PBCV-1 genome identified as modified using Pacbio's DNA modification analysis tools. (Coverage: 255X)

Replicate	motifString	modificationType	fraction	nDetected	nGenome	meanScore	meanIpdRatio	objScore
PBCV1-1C	CATG	m6A	0.98612654	1777	1802	326.93134	5.298204	573698.06
PBCV1-1C	GATC	m6A	0.870283	1476	1696	297.26627	4.273591	387192.03
PBCV1-1C	DTNRRDDDG	modified_base	0.17467625	607	3475	46.228996	1.6845644	5835.9775
PBCV1-1C	TNNNDNNH	modified_base	0.11900781	15089	126790	45.164227	1.6761321	100339.734
PBCV1-1C	TNNNCRVH	modified_base	0.08545584	1077	12603	42.325905	1.6427859	4981.8374
PBCV1-2A	MNNGANGCAGYA	m6A	1	12	12	141.16667	1.7216667	1694
PBCV1-2A	CATG	m6A	0.9889012	1782	1802	328.59653	5.322953	579706.6
PBCV1-2A	GATC	m6A	0.8832547	1498	1696	300.22498	4.2772593	402194.38
PBCV1-2A	TNNNDNNH	modified_base	0.11322659	14356	126790	44.60365	1.674846	90148.28
PBCV1-2A	TNVRDDDG	modified_base	0.11033353	741	6716	43.268555	1.6505132	4409.886
PBCV1-2A	TNNNCRVH	modified_base	0.07934619	1000	12603	42.278	1.6454105	4322.0312
PBCV1-E1	TNAGAGTTNKNNNNNNG	m6A	1	5	5	76.6	1.464	383
PBCV1-E1	DNTNNGCATAANT	modified_base	1	10	10	48.6	1.7650001	486
PBCV1-E1	CATG	m6A	0.98612654	1777	1802	324.51773	5.2549305	569462.6
PBCV1-E1	WNNNNNGANGCAGCA	m6A	0.9166667	11	12	139.36363	1.6345454	1417.5306
PBCV1-E1	WGAGGCNNNTNYA	m6A	0.875	7	8	89.28571	1.4628571	554.2265
PBCV1-E1	ANNKNTNTNNGCNTNNTT	modified_base	0.875	7	8	46.57143	1.6557142	289.08453
PBCV1-E1	GATC	m6A	0.8649764	1467	1696	298.743	4.2358856	384619.88
PBCV1-E1	TNNNAGTTNGNANTNNNT	m6A	0.85714287	6	7	69.5	1.6666666	362.98105
PBCV1-E1	HNNNNNAGGCMNTTG	m6A	0.85714287	6	7	90.333336	1.6550001	471.7883
PBCV1-E1	TNACGANAANTNNNNNA	m6A	0.8333333	5	6	97.4	1.5059999	413.3004
PBCV1-E1	TNNNTTGANNNAGNNNTG	m6A	0.8333333	5	6	86.6	1.7739999	367.47244
PBCV1-E1	ANANNNAGNGNGNNAYT	m6A	0.75	6	8	78.5	1.7916666	363.55997
PBCV1-E1	AGAGAAWAA	m6A	0.75	6	8	103	1.6749998	477.0277
PBCV1-E1	ANANTNANAGANNANNY	m6A	0.72727275	8	11	61.375	1.5675	368.64563
PBCV1-E1	YNNAGGNWAAANT	m6A	0.6666667	8	12	64	1.62	355.45764
PBCV1-E1	TNNNNNNASYTASTA	m6A	0.6666667	10	15	79.4	1.8400002	551.23706
PBCV1-E1	ANNNNTNAGNAAAAA	m6A	0.57894737	11	19	78.63636	1.4945455	528.9216
PBCV1-E1	GNANNNNHANNTGGCA	m6A	0.5714286	8	14	75.5	1.58875	365.00827
PBCV1-E1	AGNAAATTTT	m6A	0.5	7	14	82.28571	1.8385714	308.67075
PBCV1-E1	ADKYAGYANY	m6A	0.41666666	40	96	127.825	2.0889997	1133.237
PBCV1-E1	TNRADRRG	modified_base	0.27981222	298	1065	45.78859	1.6870131	4336.642
PBCV1-E1	TNNNDNNH	modified_base	0.11198833	14199	126790	44.66702	1.6770811	88409.766
PBCV1-E1	TNNNCRVH	modified_base	0.08045703	1014	12603	42.001972	1.6423571	4408.7476
PBCV1-E1	TVNNNDDG	modified_base	0.061388757	1022	16648	42.930527	1.6568396	2088.423

Note: Pacbio advertises 25X as sufficient coverage to detect all real m6A modifications in a sample sequence

Table S4C. DNA sequences in the PBCV-1 genome identified as modified using Pacbio's DNA modification analysis tools. (Coverage: >1200X)

Replicate	motifString	modificationType	fraction	nDetected	nGenome	meanScore	meanIpdRatio	objScore
PBCV1-1C	CATG	m6A	0.9916759	1787	1802	1318.0654	5.2829576	2337729.8
PBCV1-1C	GATC	m6A	0.9817217	1665	1696	988.5249	4.248782	1618793.4
PBCV1-1C	AGDVAAAAW	m6A	0.4939759	82	166	259.4878	1.4247562	11278.88
PBCV1-1C	TNNNNNNH	modified_base	0.16766186	26506	158092	71.82713	1.5380374	381613.38
PBCV1-1C	TNNNDNDG	modified_base	0.09227239	2252	24406	66.023094	1.5094546	17411.205
PBCV1-2A	CATG	m6A	0.9916759	1787	1802	1410.751	5.3039694	2502117.5
PBCV1-2A	GATC	m6A	0.9829009	1667	1696	1047.8032	4.2681665	1719784.8
PBCV1-2A	AGDVAAAAW	m6A	0.4759036	79	166	273.10126	1.4377215	11059.052
PBCV1-2A	TNNNNNNH	modified_base	0.16515067	26109	158092	71.32479	1.5295854	368233.3
PBCV1-2A	TNRVNNDG	modified_base	0.16131958	1423	8821	67.71468	1.509661	18655.469
PBCV1-2A	DNNNTGYNNNNG	modified_base	0.1509329	542	3591	69.89299	1.530037	6907.7783
PBCV1-E1	GNNANNTNGCANTNNCA	m6A	1	6	6	343.83334	1.5016667	2063
PBCV1-E1	ANNAGANNNAGCAA	m6A	1	7	7	268.7143	1.5757143	1313.5214
PBCV1-E1	AATGANGAANNNT	m6A	1	6	6	344	1.4133333	2064
PBCV1-E1	CATG	m6A	0.9900111	1784	1802	1203.7657	5.2254577	2128202
PBCV1-E1	GATC	m6A	0.9811321	1664	1696	919.5919	4.2007504	1504191.8
PBCV1-E1	AGNVAAAAWH	m6A	0.48125	77	160	234.22078	1.4315586	9337.913
PBCV1-E1	TNNNNNNH	modified_base	0.16286087	25747	158092	69.2657	1.5390226	348241.03
PBCV1-E1	TNRVNNDG	modified_base	0.15905227	1403	8821	64.91661	1.5147673	17410.023
PBCV1-E1	TGYNNNNG	modified_base	0.13299957	611	4594	64.89689	1.5278397	6452.534
PBCV1-E1	AGKNNNNH	m6A	0.054108746	615	11366	144.39024	1.4278698	5613.9644

Note: Pacbio advertises 25X as sufficient coverage to detect all real m6A modifications in a sample sequence

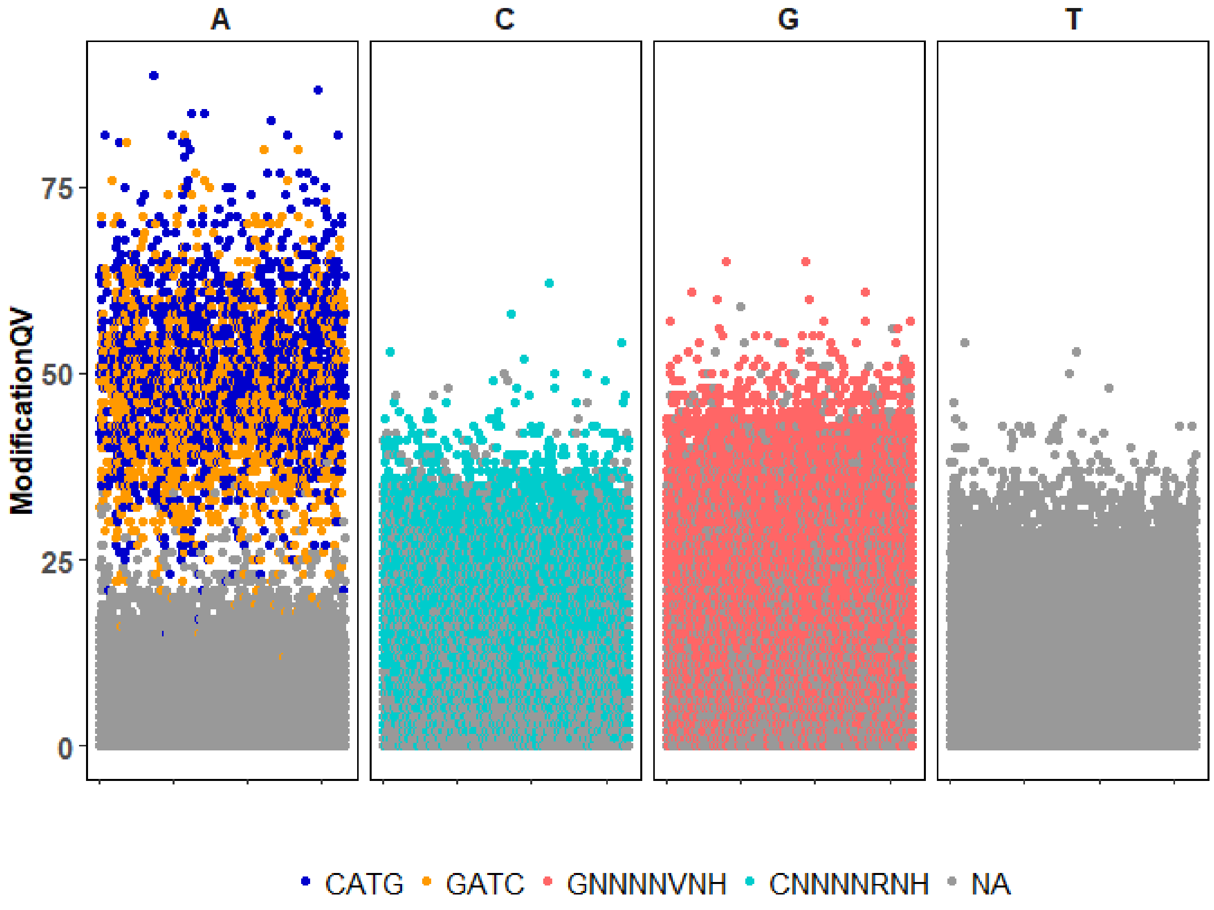

Figure S4. Average ModificationQV scores for all nucleotides in the PBCV-1 genome at 30-fold read recruitment coverage. Dot color denotes association with a motif detected by motifMaker.sh. Motifs not detected in all three replicates are not shown.

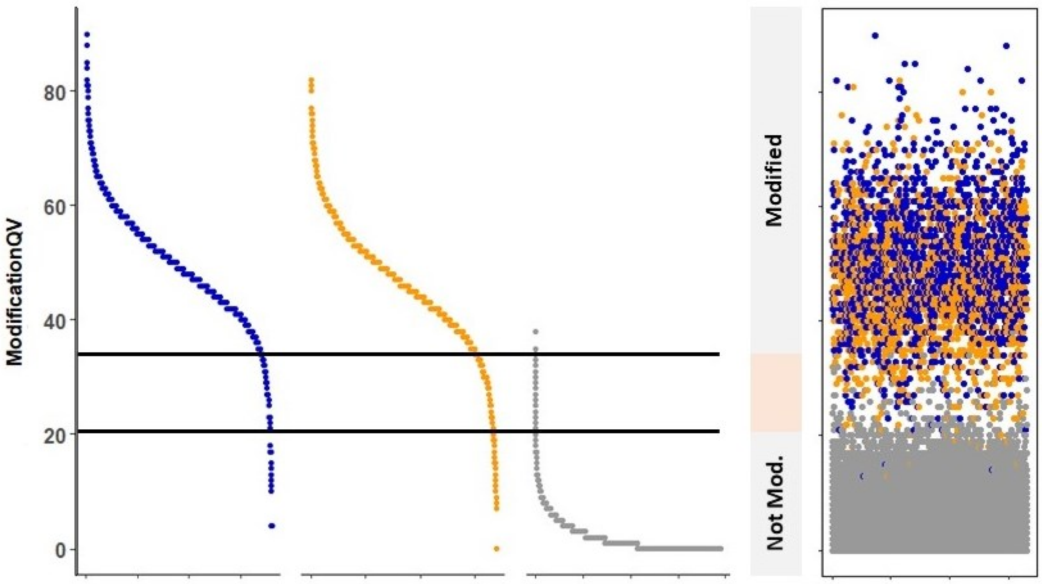

Figure S5. Re-visualization of PBCV-1 adenines as a rank ordered distribution of the ModificationQV score at a read-recruitment coverage of 30-fold. Boundaries have been overlaid to demonstrate how one could confidently identify modified and non-modified sites, yet, there is an unclear region wherein the modification status is uncertain. Thus, we used the default ModificationQV value of 30 as a threshold for deciding modification status. Blue dots denote adenines in CATG contexts, and orange dots denote adenines in GATC contexts.

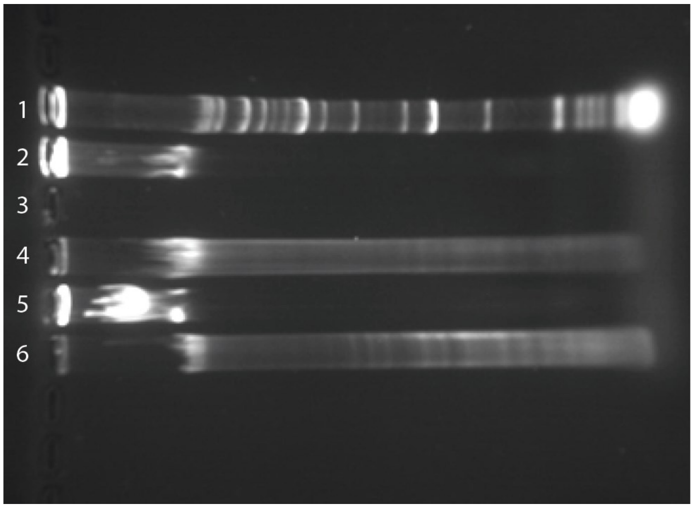

Figure S6. Restriction digestion analysis of Chlorovirus PBCV-1 genomic DNA. 1) 40kb Extension Ladder; 2) PBCV-1 genomic DNA; 3) Loading Buffer; 4) PBCV-1 DNA + DpnI; 5) PBCV-1 DNA + DpnII; 6) PBCV-1 DNA + Sau3AI.
